# Exploring the effects of short-term forest bathing on anxious medical undergraduates’ stressful emotions using near-infrared functional brain imaging and facial expression technology

**DOI:** 10.3389/fpsyg.2026.1734650

**Published:** 2026-03-11

**Authors:** Rongshan Tao, Dingfeng Long, Ju Zhang, Ruiyu Long, Yu Cao

**Affiliations:** 1 School of Public Health, The key Laboratory of Environmental Pollution Monitoring and Disease Control, Ministry of Education, Guizhou Medical University, Guiyang, China; 2 Guizhou Prenatal Diagnosis Center, The Affiliated Hospital of Guizhou Medical University, Guiyang, China

**Keywords:** enrolled medical undergraduates, functional near-infrared spectroscopy, oxyhemoglobin, short-term forest bathing, stress relief

## Abstract

**Background:**

Enrolled medical undergraduates face high exam competition and stress, reducing life quality, well-being, learning abilities and health; supporting their exam stress management is critical. This study used a 2-h forest bathing intervention to alleviate their exam-related stress.

**Methods:**

One week prior to the intervention, 160 students were recruited via university bulletin boards; eligibility screening, defined by a State–Trait Anxiety Inventory-State score >40, was administered 1 day before the 2-h sensory exposure. Ultimately, 60 final-examination-preparing medical undergraduates (male:female = 7:53) were randomly assigned to two groups (*n* = 30 per group). Analyses focused on oxygenated hemoglobin (oxy-Hb) levels in the prefrontal cortex (PFC), physiological functions, and psychological changes under specific tasks.

**Results:**

Forest environments characterized by lower noise levels, and higher negative air ion concentrations—with a comfort index of 0.971–1.368 (vs. 2.221–3.647 in urban areas) and negative oxygen ion concentrations of 1,000–1,200 ions/cm^3^ (vs. 400–500 ions/cm^3^ in urban areas). For the PFC region, forest group had significantly greater oxy-Hb reduction during the Trier Social Stress Test (TSST) [*t*(29) = 3.038, *p* = 0.005]. At the channel-specific level, forest group had decreased oxy-Hb in the left PFC and bilateral Frontopolar Area during the TSST, while the urban group exhibited increased oxy-Hb in the right PFC during the MT. No between-group oxy-Hb differences were observed in the Stroop task or rumination task. Forest group showed lower heart rate [*F*(1,57) = 4.227, *p* = 0.044], salivary cortisol [*F*(1,57) = 4.590, *p* = 0.036], higher Nature Connection Scale [*F*(1,57) = 4.822, *p* = 0.032], Digit Span Backward Test [*F*(1,57) = 6.164, *p* = 0.016], Montreal Cognitive Assessment scores [*F*(1,57) = 12.040, *p* < 0.001], lower Rumination-Reflection Questionnaire [*F*(1,57) = 11.318, *p* = 0.001]/Perceived Stress Scale scores [*F*(1,57) = 6.076, *p* = 0.017], 56.7% positive facial expressions (*U* = 263.000, *n* = 60, *p* = 0.002), and elevated positive affect [Profile of Mood States: *F*(1,57) = 17.063, *p* < 0.001].

**Conclusion:**

Short-term forest bathing reduces physiological stress markers, enhances nature connectedness/positive emotions, alleviates stress via modified cerebral blood flow, and improves memory and reduce cognitive fatigue to a certain extent. As a low-cost, easy-to-implement strategy, it is may recommended for enrolled medical undergraduates’ mental health curricula to build sustainable stress management.

## 1 Introduction

Medical undergraduates face greater mental health challenges than other students, due to extensive professional knowledge, rigorous skill assessments, and their future profession’s link to human life and health (Memon et al., 2023). Academic assessments often serve as one of the major stressors for this group, as success in these evaluations is closely linked to their future career trajectories (Afrisham et al., 2016). Owing to the unique nature of the medical profession, medical students often exhibit strong professional responsibility and a keen desire for learning, and experience elevated stress during academic assessments (Bergmann et al., 2019). Negative emotions (e.g., anxiety, stress) are often associated with impaired clinical performance (Xi et al., 2025), but may in some contexts enhance certain aspects of performance (Boscolo et al., 2025; LeBlanc, 2009; Xi et al., 2025). While moderate stress facilitates academic achievement, persistent stress impairs cognition and emotion— critical precursors to clinical practice competence and key to their professional development (Arnsten, 2009; LeBlanc, 2009; Leblanc et al., 2012; Tam et al., 2025; Woo et al., 2021). Evidence indicates that emotional intelligence (EI) exerts effects on both perceived stress and clinical practice-related performance (Hussain and Burdey, 2023; Zhang et al., 2025). Specifically, EI buffers stress-induced adverse effects for medical learners: individuals with higher EI better regulate stress responses, maintaining consistent performance across clinical tasks from diagnostic reasoning to patient communication (Horne et al., 2024). While EI can be enhanced via training, and its efficacy in improving individual emotional well-being has been validated, it is geared toward long-term competency development. Coupled with marked heterogeneity in stress triggers across different groups, strategies for alleviating stress in this population warrant further investigation.

An increasing body of research has shown the stress-relieving efficacy of forest exposure (Kang and Shin, 2020; Kweon et al., 2024). Forests, a crucial component of natural settings, benefit medical undergraduates’ physiological health (Song et al., 2014) and alleviate negative emotions like anxiety and depression (Morita et al., 2007). Leveraging forest-based interventions to support the physical and mental health of medical undergraduates could represent a significant method in addressing these concerns. Forest bathing, a nature-based therapy, entails purposeful exposure to forest environments, breathing in beneficial elements, and immersing in the landscapes, atmospheres, and sounds of the forest; it improves physical and mental health (Li et al., 2022). Research indicates forest bathing decreases blood pressure, boosts the autonomic nervous system and immune function, alleviates depression, and fosters well-being (Antonelli et al., 2019; Li et al., 2022; Queirolo et al., 2024; Vermeesch et al., 2024).

Most current forest bathing studies focus on extended cycles (Bang et al., 2017). For exam-preparing medical undergraduates, perceived stress usually fades fast after exams. Additionally, due to constraints like conflicting academic schedules, spatial distance, and transportation limitations, long-term forest bathing reduces their subjective willingness to participate. This creates challenges in differentiating intervention effects and maintaining sample integrity, thereby rendering long-term forest bathing generally impractical to implement during this period. Recent studies indicate a single session of short-term forest bathing (15 min to 2 h) significantly improves cardiorespiratory function and induces physiological and psychological relaxation (Song et al., 2019; Vermeesch et al., 2024). The positive effects of short-term forest bathing—encompassing walking, viewing scenery, and listening to ambient sounds—on human health have been increasingly investigated. Beyond improvements in cardiopulmonary function, its potential to optimize brain function has gradually emerged. As the body’s central organ, the brain’s activity directly influences stress, emotion, and cognition (Puxeddu et al., 2022). The hypothalamus and hippocampus are key regulators of the hypothalamic–pituitary–adrenal (HPA) axis that mediates stress responses, and perceived stress levels are modulated by these structures (Herman, 2012). Perceived stress is also modulated by altered functional connectivity within the amygdala-prefrontal cortex, and dysregulation of this integrated neural circuitry represents the core mechanism of stress-related disorders (Caetano et al., 2022). Sudimac et al. (2022) reported that a 60-min forest walk intervention correlated with decreased amygdala activity, which interpreted as signaling attenuated stress responses and enhanced control over negative emotions linked to anxiety and fear.

Current neuroscience technologies encompass functional magnetic resonance imaging (fMRI) and functional near-infrared spectroscopy (fNIRS) (Wilcox and Biondi, 2015). Compared to fMRI, fNIRS presents advantages such as portability, lower noise levels, and fewer physical constraints, making it more acceptable to participants and easier for researchers to utilize (Pinti et al., 2020). fNIRS employs infrared technology to monitor real-time changes in cerebral cortical oxygenation, tracking concentrations of oxygenated hemoglobin (oxy-Hb) and deoxygenated hemoglobin (deoxy-Hb) in the cerebral cortex, thus mapping hemodynamic changes in localized brain areas (de Faria et al., 2020). Research shows changes in oxy-Hb correspond with cerebral blood flow alterations, and reduced oxy-Hb concentration correlates with stress relief (Ohtani et al., 2009). When stress is alleviated, activity in localized brain regions decreases, along with cerebral blood flow and oxy-Hb levels (Ikei et al., 2018). For example, Jo et al. (2019) utilized fNIRS to demonstrate auditory stimulation with forest sounds decreases oxy-Hb concentration in the right prefrontal cortex, inducing relaxation. Similarly, Yamashita et al. (2021) observed lower oxy-Hb levels in the right orbitofrontal cortex after exposure to forest images, accompanied by improved mood. In recent years, facial expression recognition technology has been extensively applied in facial expression studies (Nejati et al., 2022). For instance, Fu et al. (2023) evaluated facial expression imitation differences to facilitate depressed patients’ diagnosis. To date, limited research has applied fNIRS combined with facial expression recognition technology to explore short-term forest bathing’s effects on medical undergraduates’ physiological and psychological responses.

In summary, this study will employ fNIRS and facial expression recognition to measure changes in oxy-Hb levels and facial emotional expression in medical undergraduates before and after short-term forest bathing. It will primarily explore the effects of this intervention on the group’s stress-related emotions by integrating multidimensional physiological and psychological indicators; concurrently, it will monitor forest environmental factors, providing empirical data to assess forest comfort and elucidate the intervention’s underlying mechanism.

## 2 Objects and methods

### 2.1 Participants

Sample size was estimated via *a priori* power analysis using G*Power 3.1.9.7 (Heinrich-Heine-Universität Düsseldorf), anchored to the primary outcome: prefrontal cortex (PFC) oxyhemoglobin (HbO) concentration changes during the Trier Social Stress Test. The analysis was based on between-group comparisons (experimental vs. control groups) using independent-samples *t*-tests, with the following parameters: *α* = 0.05, statistical power (1-*β*) = 0.80, assumed effect size Cohen’s *d* = 0.8 [supported by typical effect sizes of PFC HbO changes in similar fNIRS studies; (Int-Veen et al., 2023; Jo et al., 2019)], and a two-tailed test. The power analysis indicated a minimum required sample size of 52 participants. Participants were screened using inclusion and exclusion criteria, with exclusions based on these criteria.

Inclusion criteria:

1. State Anxiety Inventory (STAI-S) score > 40 (indicating potential level of test-related anxiety) (Thomas and Cassady, 2021);
2. Ability to voluntarily participate and complete study-related tasks.

Exclusion criteria:

1. Psychiatric disorders or comorbidities;
2. Personal history of brain surgery or head trauma;
3. Females during menstruation.

To identify stressed enrolled medical undergraduates and ensure statistical power, a pilot survey was conducted based on the study by Macauley et al. (2018), which revealed that approximately 39.6% of medical students scored above 40 on the STAI-S during the revision period of the exam month. A total of 160 students were recruited via bulletin boards at a medical university in Guizhou Province 1 week prior to the intervention. One day before the intervention, all 160 recruits completed the STAI-S for eligibility screening, with 65 students meeting the criterion of STAI-S > 40. Subsequently, five participants were excluded due to voluntary withdrawal, yielding a final sample of 60 participants for all statistical analyses (19.633 ± 0.974 years; male:female = 7:53). All participants provided written informed consent before enrollment and could withdraw at any time if unwell. The study was conducted in accordance with the Declaration of Helsinki guidelines, and the protocol was approved by the Ethics Committee of a medical university in Guizhou Province (approval ID: 2025234).

### 2.2 Study design

**Figure 1:**
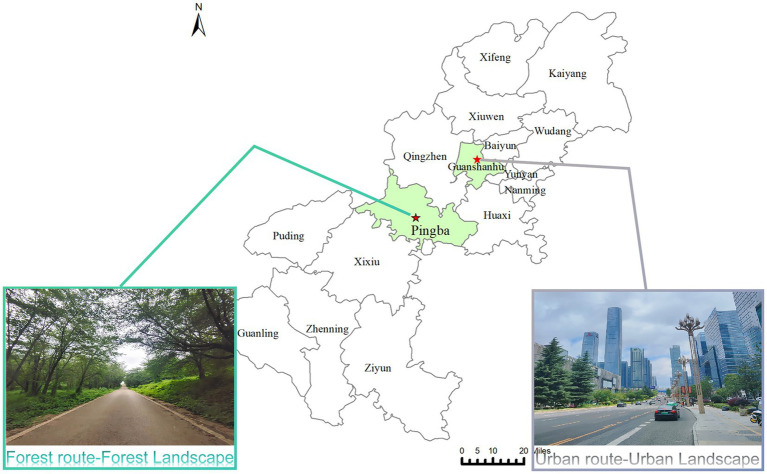
Locations of the nature and urban walks. Map lines delineate study areas and do not necessarily depict accepted national boundaries.

The Pingba Forest Base, upstream of Hongfeng Lake in Gui’an New Area, covers approximately 1,600 hectares and has a subtropical monsoon climate. Its core environmental parameters are as follows: average elevation 1,250 m, mean annual temperature 17.5 °C, annual precipitation 1,298 mm, mean tree age of 20 years, average tree height 3–5 m, stocking density 437.5 trees/hectare, and light intensity 2,500–3,500 lx. Dominant tree species include cherry blossoms, tea plants, camphor trees, sweet osmanthus, and red maples, forming a mixed vegetation structure of “cherry blossom-tea-broadleaf tree.” It is a natural peri-urban forest environment conducive to forest bathing. Guiyang North Railway Station, in Guanshanhu District, Guiyang City, Guizhou Province, is an urban transportation hub surrounded by urban buildings and major transportation routes, embodying a typical urban setting. Accordingly, the forest group in this study was tested at Pingba Forest Base, and the urban group at Guiyang North Railway Station (see Figure 1).

**Figure 2:**
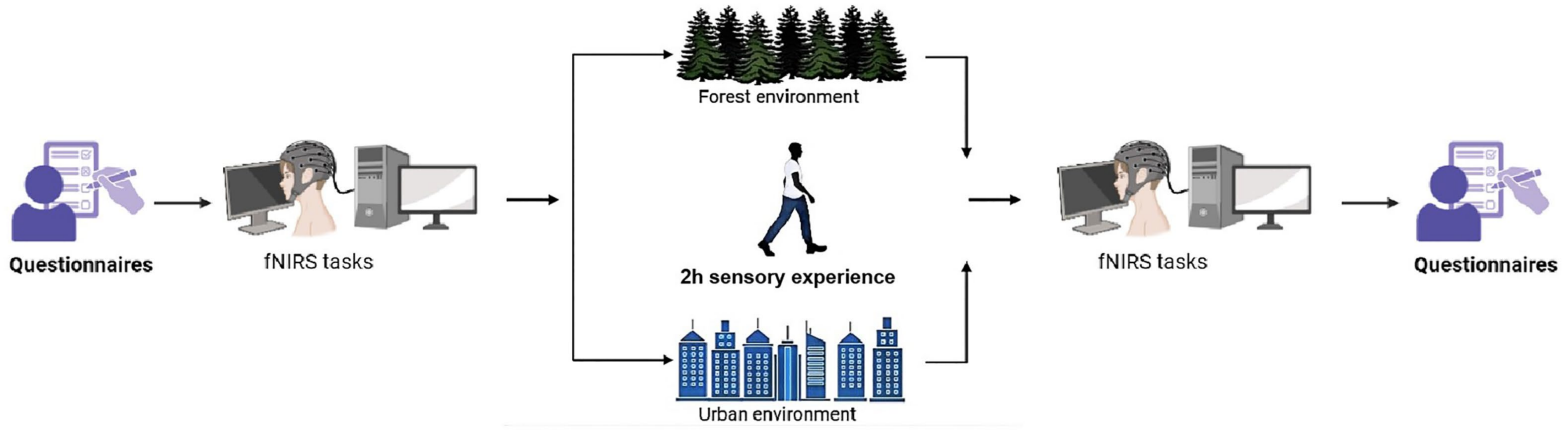
Flowchart of the study procedure.

This study utilized a mixed design that combined within-subjects pre-post comparisons with between-group comparisons to assess the impact of short-term forest bathing on medical undergraduates’ brain activity and various physical and psychological indicators. The experiment took place in July, with same-day measurements of negative air ions (NAIs), microclimate (air temperature, relative humidity, wind speed, and noise levels), and volatile organic compounds (VOCs). Participants were randomly assigned to either the forest or urban walking groups using a random number table. The forest group’s intervention included 15-min Forest Acclimation and Sensory Priming, 60-min Forest Multisensory Exploration, 30-min Forest Meditation and Connection, and 15-min reflection; the urban group received 15-min Urban Setting Adaptation and Warm-up, 60-min Urban Multisensory Walk, 30-min Urban Relaxation and Reflection, and 15-min debriefing. All participants knew they would take part in a fNIRS study but not the specific protocol (Song et al., 2019; Song et al., 2018; Sudimac et al., 2022). On the day before the intervention, 160 recruited participants underwent STAI-S screening at 8:00 a.m. at a medical university in Guizhou Province. Of the 65 eligible participants, 5 withdrew voluntarily; fasting salivary cortisol samples were collected from the final 60 participants at 10:00 a.m., followed by questionnaire surveys, physiological parameter assessments (blood pressure, heart rate, and blood oxygen saturation), and fNIRS scanning. At 10:00 a.m. the following day, they proceeded to the testing site and underwent a 2-h intervention along the predetermined route in their respective environments under researchers’ guidance. Throughout the walk, facial photographs were taken. Post-intervention (at 12:00), participants returned to the university for collection and testing of the same indicators (see Figure 2). Participants were instructed to abstain from smoking, alcohol, and functional beverages 24 h prior to and throughout the study. Additionally, 2 days before the intervention, they were instructed to maintain an 8-h or longer fast overnight.

### 2.3 Study measurement

#### 2.3.1 fNIRS

Changes in oxy-Hb levels in the PFC were analyzed using an 8 × 7-channel fNIRS system (NIRSport, NIRX Medical Technologies, Germany), with a probe spacing of 3 cm and a sampling rate of 7.81 Hz. Light sources and detectors formed 20 measurement channels, which were positioned over four distinct PFC subregions: the dorsolateral prefrontal cortex (DLPFC), frontopolar area (FPA), ventrolateral prefrontal cortex (VLPFC), and orbitofrontal cortex (OFC) (Chen, 2022) (see Supplementary Table S1 and Supplementary Figure S1). Data were processed using the data preprocessing module of NirsLAB software (Pfeifer et al., 2018; Ramacciotti et al., 2024; Xu and Barbour, 2016): Prior to further preprocessing, channels with a coefficient of variation (CV) > 15% were excluded due to poor signal quality; extraneous time segments were discarded; a motion artifact correction algorithm was applied via the “Remove Discontinuities” function—specifically, a fixed 1-s moving window was utilized for artifact detection, which was slid across the signal with a step size of 1 sampling point (≈0.13 s, corresponding to the 7.81 Hz sampling rate), and a 5SD threshold (default value of nirsLAB) was applied within each window to identify motion-contaminated points (Xu and Barbour, 2016), with linear interpolation adopted for imputation of the identified contaminated data points; a 0.01–0.2 Hz bandpass filter was then used to eliminate unwanted frequency signals, thereby attenuating motion-, respiratory-, and cardiac-related components while preserving signals relevant to fNIRS (see Supplementary Figure S2); and the measured optical data were converted to oxy-Hb data via the modified Lambert–Beer law.

Four tasks were designed via E-Prime 2.0: Ruminative Task (RT) and Trier Social Stress Test (TSST) to examine stress reduction via short-term forest bathing, and Memory Task (MT) and Stroop Task (ST) to assess effects on memory and cognitive function. Pre- and post-intervention, task difficulty was equated across participants, with the presentation order of tasks and stimuli randomized. Participants were instructed to undergo baseline measurement of resting oxy-Hb concentration 1 day pre-test. To prevent excessive artifacts and drifts, all participants were required to secure their heads with a head fixation device, sit upright while wearing the fNIRS device, and refrain from excessive movements (e.g., head shaking or leg movements) throughout all tasks. Participants were instructed to press the spacebar to initiate each task, after which they pressed the spacebar again to enter the practice trials. Once they understood the task procedure, they pressed the “Start Test” button for formal trials. A red “+” appeared to cue the upcoming presentation of the next trial. After completing each task, participants were instructed to close their eyes and relax for 30 s before proceeding to the next (see Supplementary Material 2).

#### 2.3.2 Physiological measurements

Blood pressure/heart rate were measured via a digital sphygmomanometer (HEM-7124, Omron Healthcare (Dalian) Co., Ltd., Dalian, China), oxygen saturation via a pulse oximeter (CMS50D, Contec Medical Systems Co., Ltd., Qinhuangdao, China). Salivary cortisol samples were collected using a Salivette^®^ device (51.1534.500, Zhuocai Biotechnology Co., China). Salivary cortisol concentrations were quantified using an enzyme-linked immunosorbent assay (ELISA kit; Wuhan Fine Biotech Co., Ltd.).

#### 2.3.3 Psychological measurements

Psychological assessments used: Connectedness to Nature Scale (CNS) (Lovati et al., 2023), Perceived Stress Scale (PSS) (Yılmaz Koğar and Koğar, 2024), Rumination-Reflection Questionnaire (RRQ) (Trapnell and Campbell, 1999), Digit Span Backward (DSB) task (Ostrosky-Solís and Lozano, 2006), Montreal Cognitive Assessment (MoCA) (Nasreddine et al., 2005), and Profile of Mood States (POMS) with Chinese normative data (Zhu, 1995). Facial Expression Recognition: Anaconda3 (Anaconda Distribution; Anaconda, Inc., Austin, TX, US) was employed to classify captured photographs into seven emotions: anger, disgust, fear, happiness, sadness, surprise, and calmness.

#### 2.3.4 Forest environmental measurements

Air temperature and relative humidity were measured using a thermohygrometer (testo 605-H1, Shanghai Yibo Instrument Co., Ltd., Shanghai, China); wind speed was measured with an anemometer (VelociCalc^®^ 9,535/9535A, TSI Incorporated, United States); NAIs concentration was quantified using a negative ion detector (LD-FY1, Shandong Leand Intelligent Technology Co., Ltd., China); and noise levels were measured via a digital sound level meter (CEL-246, Qingdao Lubo Weiyeh Technology Co., Ltd., Qingdao, China). VOCs were analyzed using a gas chromatography/mass spectrometry (GC/MS) system (Agilent 8,890/7000D, Agilent Technologies, Santa Clara, CA, United States). NAIs concentration was evaluated in accordance with the *Grade of Air Negative (Oxygen) Ion Concentration* (QX/T 380–2017) (China Meteorological Administration, 2017). Environmental comfort was evaluated using the Lu Dinghuang Comprehensive Comfort Index (Lv et al., 2024) (see Supplementary Material 3). As the experiment was conducted during daytime, the two sites’ acoustic environment was classified based on daytime ambient noise limits specified in *The Acoustic Environment Quality Standard* (GB 3096–2008) (General Administration of Quality Supervision, Inspection and Quarantine of the People’s Republic of China [AQSIQ] and Standardization Administration of the People’s Republic of China [SAC], 2008) (see Supplementary Table S4).

### 2.4 Statistical analysis

Data analyses used Jamovi 2.4.14 (The jamovi project, Sydney, Australia). For the analysis of pre- and post-walking data with baseline adjustment, analysis of covariance (ANCOVA) was used for between-group comparisons, with post-intervention outcomes as the dependent variable, pre-intervention baseline values as the covariate, and group as the between-subjects factor; paired t-tests were applied for within-group comparisons (pre- vs. post-walking). Effect sizes for ANCOVA were reported using partial eta squared ( $\eta_{p}^{2}$ ). Pre-intervention outcomes were presented as mean ± standard deviation (M ± SD), while baseline-adjusted outcomes were reported as mean ± standard error (M ± SE). For fNIRS data analysis, the mean oxy-Hb values across the 20 measurement channels were used for subject-level analyses (cross-channel aggregation); for channel-specific analyses (as exploratory analyses), the false discovery rate (FDR) method was employed to correct *p*-values, thereby reducing the occurrence of false positive results. Normality and variance homogeneity were evaluated via Shapiro–Wilk test and Levene’s test, respectively. Pearson correlation analysis was applied to normally distributed continuous variables, while Spearman rank correlation analysis was used for non-normally distributed or ordinal variables. Facial expression data were analyzed using the Mann–Whitney *U* test. A two-tailed significance level of *α* = 0.05 was adopted for all analyses.

## 3 Results

### 3.1 Demographic characteristics

Sixty participants were enrolled; the two groups had no significant demographic differences across all variables (see Supplementary Table S5).

### 3.2 Evaluation of forest environmental metrics and VOCs

**Figure 3:**
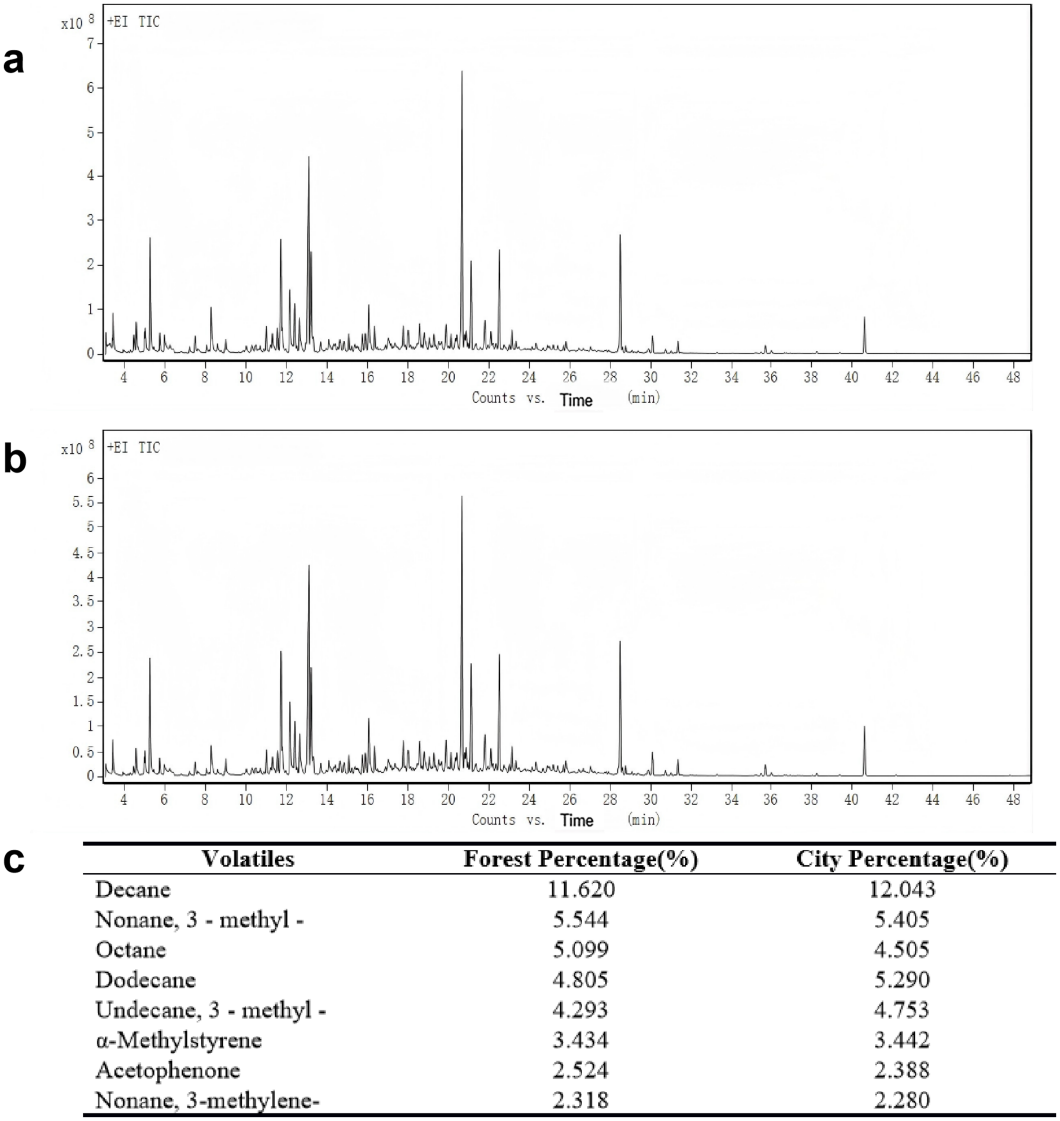
Main volatile compounds and percentage content of forest and urban ambient air. **(a)** VOCs detection results in the forest environment. **(b)** VOCs detection results in the urban environment. **(c)** Compositions of major VOCs in air of the two environments.

Environmental comfort is primarily determined by ambient temperature, relative humidity, and wind speed, with lower index values indicating a more comfortable thermal environment (Wang et al., 2025). Environmental comfort index results showed forest values 0.971–1.368 and urban values 2.221–3.647. The higher the NAIs concentration, the fresher the air (China Meteorological Administration, 2017). NAIs concentrations were higher in forest environments (1,000–1,200 ions/cm^3^) than in urban environments (400–500 ions/cm^3^). Forest NAIs corresponded to a relatively high concentration with relatively fresh air, while urban NAIs were only at a moderate concentration with moderate air quality (see Supplementary Table S3). Acoustic environment tests revealed that forest noise levels generally met the criteria for rehabilitation, convalescence [50 dB (A)], and residential areas [55 dB (A)], whereas urban noise levels reached the threshold for trunk railway areas [70 dB (A)] (see Table 1 and Supplementary Table S4). Moreover, VOC analysis revealed similar VOC composition in forest and urban air (see Figure 3).

**Table 1:** Comparison of forest and urban environments.

Environmental indicators	Forest	City
Temperature (°C)	22.6 ~ 24.2	25.9 ~ 26.6
Relative humidity (%)	75.4 ~ 76.3	78.3 ~ 79.1
Average wind speed (L/min)	1.7 ~ 3.3	3.0 ~ 4.9
Composite comfort index (S)	0.971 ~ 1.368	2.221 ~ 3.647
Minimum noise (dB)	38.4	69.3
Maximum noise (dB)	60.3	80.1
Negative oxygen (ions/CM^3^)	1,000 ~ 1,200	400 ~ 500

### 3.3 fNIRS analysis

The nirsLAB was utilized to analyze changes in cerebral oxy-Hb concentration under various tasks. For the TSST task, ANCOVA (controlling for baseline values) revealed a significant between-group difference in oxy-Hb concentration [*F*(1,57) = 4.732, *p* = 0.034, $\eta_{p}^{2}$ =0.077], with the forest group showing a significant pre-post decrease [*t*(29) = −3.038, *p* = 0.005] and no significant change in the urban group. At the channel level, the forest group had significant oxy-Hb reductions in the left VLPFC, left DLPFC, left OFC, and bilateral FPA, while the urban group showed no such differences (see Table 2 and Supplementary Table S6). For the MT task, no overall significant between-group differences were found after controlling for baseline values via ANCOVA; post-hoc channel-wise analysis showed no pre-post oxy-Hb changes in the forest group, whereas the urban group had significant increases in the right DLPFC, right FPA, and right OFC (see Table 2 and Supplementary Table S6). No significant between-group differences were observed in the RT or ST tasks (see Table 2). Additionally, partial significant between-group differences were found in pre-post oxy-Hb changes at specific cerebral channels between the two groups (see Supplementary Figure S3).

**Table 2:** Results of ANCOVAs for between-group differences in oxy-Hb changes following short-term forest bathing.

Index	Forest group		*t*	*P*	Urban group		*t*	*P*	Between-group comparison		
	Before (*M* ± SD)	After (*M* ± SE)			Before (*M* ± SD)	After (*M* ± SE)			*F*	*P*	$\eta_{p}^{2}$
TSST	0.007 ± 0.161	−0.101 ± 0.034	−3.038	0.005**	−0.009 ± 0.165	0.003 ± 0.034	0.215	0.831	4.732	0.034*	0.077
MT	−0.019 ± 0.147	0.023 ± 0.015	1.311	0.200	0.017 ± 0.125	0.047 ± 0.015	1.265	0.216	1.306	0.257	0.022
ST	0.010 ± 0.078	0.016 ± 0.009	0.277	0.784	0.0001 ± 0.068	0.010 ± 0.009	0.750	0.459	0.211	0.648	0.004
RT	0.008 ± 0.033	−0.046 ± 0.006	−1.526	0.138	0.006 ± 0.031	0.009 ± 0.006	0.350	0.729	2.609	0.112	0.044

*oxy-Hb is expressed in μM; $\eta_{p}^{2}$ denotes partial *η*^2^ *P < 0.05, **0.001 ≤ *P* < 0.01.

### 3.4 Physiological effects

Forest group had significantly lower post-intervention systolic blood pressure (SBP), heart rate, and salivary cortisol than pre-intervention [SBP: *t*(29) = 4.744, *p* < 0.001; heart rate: *t*(29) = 2.677, *p* = 0.012; salivary cortisol: *t*(29) = 2.197, *p* = 0.036], while the urban group showed no significant pre-post changes in these indicators. ANCOVA (controlling for baseline values) revealed significant between-group differences in pre-post changes in heart rate [*F*(1,57) = 4.227, *p* = 0.044, $\eta_{p}^{2}$ =0.069] and salivary cortisol [*F*(1,57) = 4.590, *p* = 0.036, $\eta_{p}^{2}$ =0.075]. No significant between-group differences were found in SBP or oxygen saturation between the two groups (see Table 3).

**Table 3:** Statistical analysis of four physiological indicators pre- and post-walk in forest vs. urban environments.

Index	Forest group		*t*	*P*	Urban group		*t*	*P*	Between-group comparison		
	Before (*M* ± SD)	After (*M* ± SE)			Before (*M* ± SD)	After (*M* ± SE)			*F*	*P*	$\eta_{p}^{2}$
SBP (mmHg)	109.567 ± 8.831	100.928 ± 1.439	4.744	<0.001***	105.933 ± 9.516	104.238 ± 1.439	1.790	0.084	2.594	0.113	0.044
DBP (mmHg)	74.000 ± 8.179	70.356 ± 1.478	1.956	0.060	71.533 ± 8.169	69.877 ± 1.478	1.140	0.263	0.052	0.820	0.001
HR (bpm)	79.167 ± 9.851	73.363 ± 1.368	2.677	0.012*	75.800 ± 9.650	77.370 ± 1.368	−0.561	0.579	4.227	0.044*	0.069
SC (nmol/L)	9.589 ± 1.094	9.181 ± 0.131	2.197	0.036*	9.283 ± 0.877	9.581 ± 0.131	1.274	0.213	4.590	0.036*	0.075
SPO_2_ (%)	98.000 ± 3.000	97.777 ± 0.235	−1.756	0.090	98.000 ± 2.000	96.986 ± 0.235	0.477	0.637	0.916	0.342	0.016

SBP, systolic blood pressure; DBP, diastolic blood pressure; HR, heart rate; SC, salivary cortisol; SPO^2^, blood oxygen saturation; $\eta_{p}^{2}$ denotes partial *η*^2^; *P < 0.05, ***P < 0.001.

### 3.5 Psychological effects

The CNS assesses emotional bond with nature (higher scores = stronger connection). ANCOVA (controlling for baseline values) revealed a significant between-group difference [*F*(1,57) = 4.822, *p* = 0.032, $\eta_{p}^{2}$ =0.078]. Forest group had significant post-stroll CNS score increase vs. pre-stroll [*t*(29) = −3.992, *p* < 0.001], while urban group showed no change.

The PSS assesses perceived stress (lower scores = lower stress, greater emotional stability) and the POMS evaluates emotional states. For PSS, scores decreased significantly in both the forest group [*t*(29) = 4.689, *p* < 0.001] and urban group [*t*(29) = 2.101, *p* = 0.044]; ANCOVA indicated a more pronounced decline in the forest group [*F*(1,57) = 6.076, *p* = 0.017, $\eta_{p}^{2}$ =0.096].

For the POMS, Total Mood Disturbance (TMD) scores decreased significantly in both groups [forest: *t*(29) = 6.898, *p* < 0.001; urban: *t*(29) = 2.291, *p* = 0.029], with a significant between-group difference via ANCOVA [*F*(1,57) = 17.063, *p* < 0.001, $\eta_{p}^{2}$ =0.230]. At the emotional dimension level, forest group showed significant improvements in tension-anxiety [T-A: *t*(29) = 5.873, *p* < 0.001], anger-hostility [A-H: *t*(29) = 2.910, *p* = 0.007], fatigue-inertia [F-I: *t*(29) = 3.051, *p* = 0.005], depression-dejection [D-D: *t*(29) = 3.874, *p* < 0.001], confusion-bewilderment [C-B: *t*(29) = 5.670, *p* < 0.001] and vigor-activity [V-A: *t*(29) = −2.659, *p* = 0.013], with no change in self-esteem. The urban group only showed reductions in tension [*t*(29) = 2.579, *p* = 0.015], anger [*t*(29) = 2.447, *p* = 0.021] and confusion [*t*(29) = 2.148, *p* = 0.040]. ANCOVA revealed significant between-group differences in improvements for tension [*F*(1,57) = 11.279, *p* = 0.001, $\eta_{p}^{2}$ =0.165], depression [*F*(1,57) = 7.149, *p* = 0.010, $\eta_{p}^{2}$ =0.111] and confusion [*F*(1,57) = 4.365, *p* = 0.041, $\eta_{p}^{2}$ =0.071].

The RRQ (two subscales: immersion, reflection) showed the forest group had significantly lower scores on both [immersion: *t*(29) = 4.407, *p* < 0.001; reflection: *t*(29) = 2.691, *p* = 0.012], while the urban group had no pre-post changes. ANCOVA indicated a significant between-group difference in the immersion subscale [*F*(1,57) = 11.318, *p* = 0.001, $\eta_{p}^{2}$ =0.166], but not in reflection.

The DSB test (short-term memory) revealed a significant score increase in the forest group [*t*(29) = −5.740, *p* < 0.001] but no change in the urban group. For MoCA (cognitive function, higher scores = better performance), the forest group had a significant post-intervention increase [*t*(29) = −6.469, *p* < 0.001] while the urban group did not; ANCOVA showed significant between-group differences [DSB: *F*(1,57) = 6.164, *p* = 0.016, $\eta_{p}^{2}$ =0.098; MoCA: *F*(1,57) = 12.040, *p* < 0.001, $\eta_{p}^{2}$ =0.174] (see Table 4).

**Table 4:** Statistical analysis of six psychological indicators pre- and post-stroll in forest vs. urban environments.

Index	Forest group		*t*	*P*	Urban group		*t*	*P*	Between-group comparison		
	Before (*M* ± SD)	After (*M* ± SE)			Before (*M* ± SD)	After (*M* ± SE)			*F*	*P*	$\eta_{p}^{2}$
CNS	50.233 ± 4.739	53.363 ± 0.685	−3.992	<0.001***	50.300 ± 5.459	51.273 ± 0.685	−1.666	0.107	4.822	0.032*	0.078
PSS	21.367 ± 4.597	16.235 ± 0.926	4.689	<0.001***	21.533 ± 4.508	19.465 ± 0.926	2.101	0.044*	6.076	0.017*	0.096
RRQ-Immersion	41.100 ± 5.695	37,239 ± 0.911	4.407	<0.001***	42.633 ± 5.209	41.594 ± 0.911	0.649	0.521	11.318	0.001**	0.166
RRQ-Reflection	39.867 ± 4.321	37.590 ± 0.810	2.691	0.012*	41.267 ± 4.571	39.276 ± 0.810	1.565	0.128	1.950	0.168	0.033
DSB	4.533 ± 1.592	6.226 ± 0.290	−5.740	<0.001***	4.500 ± 1.383	5.207 ± 0.290	−1.948	0.061	6.164	0.016*	0.098
MoCA	24.733 ± 2.288	27.765 ± 0.289	−6.469	<0.001***	25.500 ± 1.393	26.335 ± 0.289	−2.322	0.027*	12.040	<0.001***	0.174
TDM	107.800 ± 15.491	92.195 ± 1.502	6.898	<0.001***	107.233 ± 14.347	100.972 ± 1.502	2.291	0.029*	17.063	<0.001***	0.230
T-A	6.033 ± 3.374	2.573 ± 0.355	5.873	<0.001***	5.700 ± 2.575	4.260 ± 0.355	2.579	0.015*	11.279	0.001*	0.165
A-H	4.567 ± 2.991	2.781 ± 0.406	2.910	0.007**	5.133 ± 3.421	3.286 ± 0.406	2.447	0.021*	0.773	0.383	0.013
F-I	5.733 ± 3.226	3.913 ± 0.498	3.051	0.005**	6.133 ± 3.026	4.921 ± 0.498	1.437	0.161	2.050	0.158	0.035
D-D	4.567 ± 2.849	2.352 ± 0.333	3.874	<0.001***	3.900 ± 2.734	3.615 ± 0.333	0.310	0.759	7.149	0.010*	0.111
V-A	11.333 ± 3.717	13.350 ± 0.749	−2.659	0.013*	10.967 ± 3.882	11.650 ± 0.749	−0.687	0.497	2.575	0.114	0.043
C-B	6.200 ± 3.316	2.801 ± 0.415	5.670	<0.001***	5.433 ± 3.126	4.032 ± 0.415	2.148	0.040*	4.365	0.041*	0.071
S-E	7.967 ± 2.593	8.742 ± 0.550	−1.246	0.223	8.100 ± 3.122	7.624 ± 0.550	0.589	0.560	2.062	0.156	0.035

T-A, tension-anxiety; A-H, anger-hostility; F-I, fatigue-inertia; D-D, depression-dejection; V-A, vigor-activity; C-B, confuse-bewildered; S-E, self-esteem; $\eta_{p}^{2}$ denotes Partial *η*^2^; *P < 0.05, **0.001 ≤ *P* < 0.01, ***P < 0.001.

The findings of the facial expression analysis indicated no significant difference in emotional states between the two groups prior to the intervention. However, post-intervention, a significant difference in emotional states was observed between the groups (*U* = 263.000, *n* = 60, *p =* 0.002). Rank-order analysis revealed that forest group exhibited a higher prevalence of positive emotions (56.7%), whereas the urban group showed a greater proportion of calm emotions (73.3%). These results indicate that short-term forest bathing reduces negative emotions and increases positive emotions (see Table 5).

**Table 5:** Statistical analysis of facial expression recognition outcomes.

Group	Emotional outcomes (before)			*U*	*P*	Emotional outcomes (after)			*U*	*P*
	Positive	Calm	Negative			Positive	Calm	Negative		
Forest	10(33.3%)	15(50.0%)	5(16.7%)	423.500	0.775	17(56.7%)	12(40.0%)	1(3.33%)	263.000	0.002**
City	7(23.3%)	20(66.7%)	3(10.0%)			5(16.7%)	22(73.3%)	3(10.0%)		

Positive emotions include happiness and surprise, negative emotions include anger, disgust, fear and sadness. **0.001 ≤ *P* < 0.01.

### 3.6 Correlation analysis

**Figure 4:**
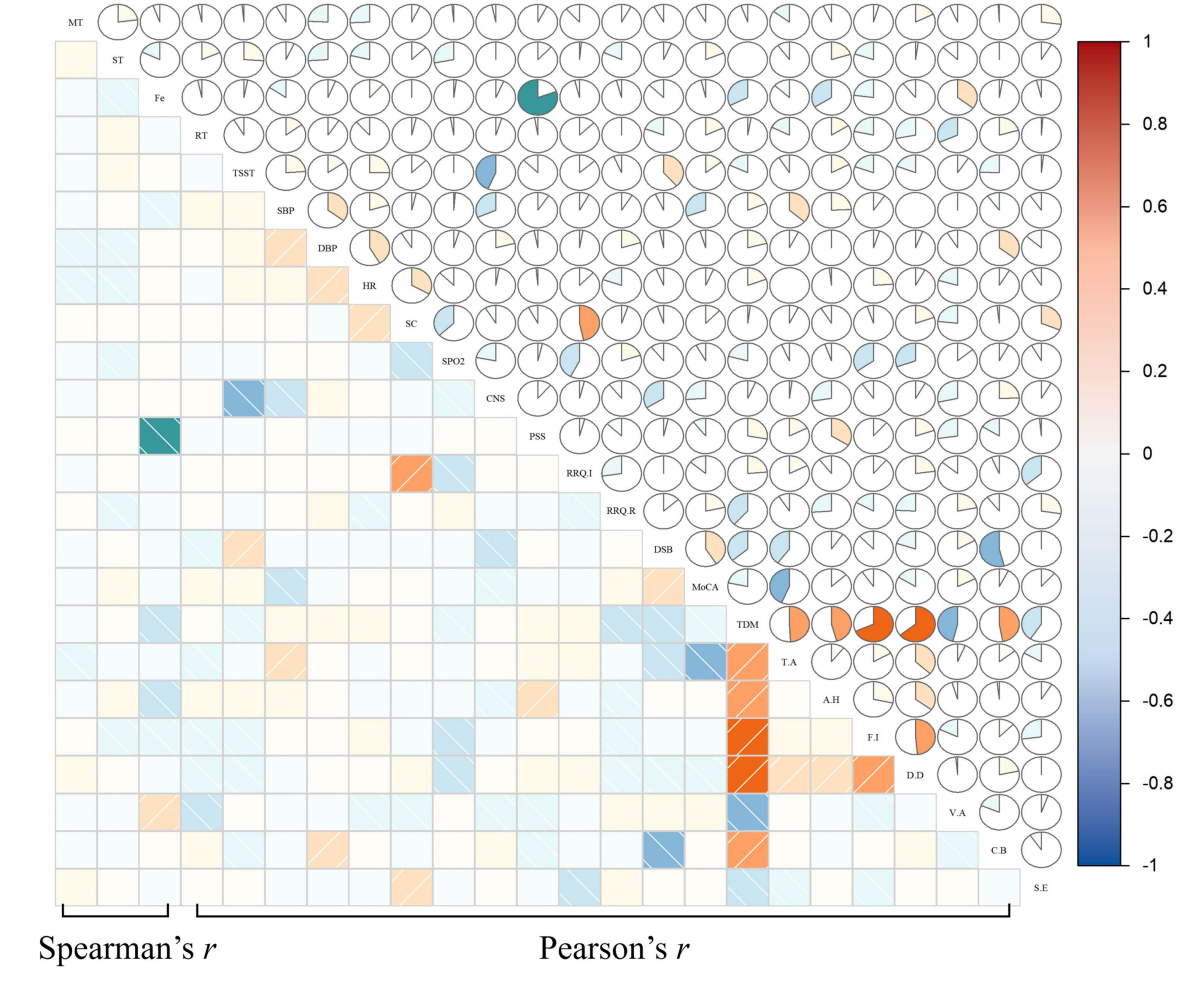
Correlation analysis of fNIRS, physiological, and psychological indices warm colors correspond to positive correlations and cool colors to negative ones; darker shades indicate stronger correlation intensity, and larger proportions in pie charts represent stronger correlations. Fe, facial expression.

Further correlation analysis was performed among the forest group for pre-to-post changes in aggregated subject-level oxy-Hb (fNIRS tasks), physiological indicators, and scale scores, as well as post-intervention facial expression data. Several significant correlations were observed: pre-to-post change in salivary cortisol was moderately positively correlated with RRQ-Immersion [*r*(28) = 0.468, *p* = 0.009], and post-intervention facial expression was positively correlated with V-A [*r*(28) = 0.369, *p* = 0.045]. Facial expression also showed a strong negative correlation with PSS [*r*(28) = −0.752, *p* < 0.001]. Meanwhile, pre-to-post changes in TSST, MoCA, and DSB were moderately negatively correlated with CNS [*r*(28) = −0.496, *p* = 0.005], T-A [*r*(28) = −0.435, *p* = 0.016], and C-B [*r*(28) = −0.539, *p* = 0.002], respectively (see Figure 4 and Supplementary Table S7).

## 4 Discussion

In today’s fast-paced, high-stress university landscape, the physical and mental well-being of medical undergraduates has increasingly emerged as a prominent concern. Prior research has established that exposure to natural environments—particularly forest settings—exerts substantial effects on stress reduction, mood enhancement, and cognitive restoration (Jimenez et al., 2021; Li, 2022; Takayama et al., 2022). Currently, most forest bathing interventions are administered continuously, with durations ranging from 3 to 7 days to even 1 month (Mao et al., 2012; Zeng et al., 2020). Nevertheless, some studies have indicated that even short-term exposure to forest environments—as brief as 15 min—can confer beneficial effects on physical and mental health (Vermeesch et al., 2024).

To investigate how short-term forest bathing alleviates psychosocial stress, we used fNIRS to measure cerebral oxy-Hb changes during stress-inducing tasks (e.g., TSST) and linked these to self-reported measures of rumination and mood. Prior work shows that acute psychosocial stress increases prefrontal cortical oxy-Hb levels to meet elevated energy demands (Bryan, 1990; Yamaoka et al., 2022), while stress alleviation is associated with reduced oxy-Hb (Burrage et al., 2018). Moreover, ruminative thinking—linked to anxiety and depression—correlates with sustained activation of the DLPFC and FPA during stress (Aldao et al., 2010; Rosenbaum et al., 2020). This provides a neural framework for testing whether forest bathing can mitigate stress-related prefrontal activation and rumination. Consistent with this framework, TSST results revealed that forest bathing significantly reduced oxy-Hb concentrations in key prefrontal regions—left VLPFC, DLPFC, OFC, and bilateral FPA (all *p* < 0.05)—in the forest group, while no such changes occurred in the urban control group. Parallel to these neural changes, the forest group also showed a marked reduction in RRQ-immersion scores (a measure of ruminative thinking; *p* < 0.001), with no significant change in the urban group. These findings link forest bathing to alleviate psychosocial stress and diminished rumination. These results align with prior evidence that natural environments mitigate rumination: Bratman et al. (2015) similarly found that forest exposure reduces prefrontal activity linked to rumination. Regarding this phenomenon, Marselle et al. (2019) proposed that green spaces reduce ruminative tendencies, while Bray et al. (2022) suggested that green spaces mitigate rumination by promoting positive awareness. The current study’s results for RRQ immersion scores, PSS, POMS, and facial expression recognition are more consistent with Bray’s findings. However, the lower RRQ reflection scores in the forest group compared to the urban group also support Marselle’s perspective, collectively indicating that short-term forest bathing may act through multiple pathways to alleviate negative thinking. However, the forest group only showed a non-significant decrease in oxy-Hb during the RT, and we speculate that significant individual differences in the design of RT items may have masked this potential effect (Westlund Schreiner et al., 2023), highlighting the need for more standardized tasks in future fNIRS studies of nature exposure.

Additionally, a key study finding is facial expression analysis. The facial feedback hypothesis posits that facial expressions actively modulate internal emotional experiences rather than merely reflecting them (Strack et al., 1988). Facial muscle activation transmits somatosensory signals to emotion-related brain regions (e.g., PFC), directly affecting neuroactivity and emotional intensity (Hennenlotter et al., 2009). Expression-emotion mismatches (e.g., smiling despite negative mood) can elicit emotional positivity (Söderkvist et al., 2018). Our findings showed that positive facial expressions in the forest group increased significantly while negative ones decreased significantly; correlation analysis further indicated a strong negative correlation between their facial expression scores and PSS [*r*(28) = −0.752, *p* < 0.001], and no such changes were observed in the urban group. This suggests the forest environment may trigger a positive feedback loop: environmental stimulation → facial muscle activation → positive expressions → PFC-mediated internal emotional regulation → emotional enhancement. Notably, scores on the PSS decreased in both the forest group (*p* < 0.001) and the urban group (*p* = 0.044). This phenomenon may be attributed to emotional projection bias, whereby participants’ subjective reports are susceptible to immediate emotional states: the transient psychological relaxation induced by single-day environmental exposure may generalize to global perceptions of stress, thus leading to reduced PSS scores. This observation aligns with the findings of Askim and Knardahl (2021), who reported that subjective reports can fluctuate with real-time emotional states. Additionally, forests are also rich in NAI. Studies have reported that NAI significantly reduces serotonin levels in the blood or brain, an effect associated with improved mood and alleviated neural tension (Jiang et al., 2018).

Improvements in memory and cognitive function are typically correlated with elevated cerebral oxy-Hb concentrations (Hadanny et al., 2020). In the MT, urban group exhibited a significant oxy-Hb increase in the right PFC compared with the forest group; no significant between-group differences were observed in the ST. However, DSB results revealed a significant score increase in the forest group (*t* = −5.740, *p* < 0.001), with no such changes in the urban group. Environmental psychology’s Attention Restoration Theory (ART) posits that natural environments restore attention through “soft fascination.” Specifically, forest elements—such as flowing water or swaying leaves—automatically draw attention without demanding focused concentration, thereby allowing the brain to rest. In contrast, urban stimuli like advertisements and traffic require sustained attention, exacerbating mental fatigue (Pasanen et al., 2018). Imamura et al. (2022) found that oxy-Hb concentration increases with mental fatigue. Although fNIRS data do not provide direct evidence that short-term forest bathing enhances memory capacity, the elevated oxygen demand observed in the urban group during the MT task may be linked to their heightened perceived fatigue and attentional demands, as proposed by ART. Forest bathing (shinrin-yoku) may alleviate fatigue perception and improve attentional efficiency through “soft fascination.” Correlation analysis also revealed a negative correlation (*r* = −0.539, *p* = 0.002) between DSB and C-B (where a lower score indicates improved attention efficiency). These findings collectively that brief forest bathing may enhance memory capacity to a certain extent. The ST showed no intergroup differences, which we speculate was due to relatively low task difficulty: the classic Stroop paradigm (e.g., only red/blue/green colors) used in this study reflects a low-to-moderate cognitive load (Macleod, 1992). Previous studies have reported an association between task difficulty gradients and the pattern of prefrontal oxy-Hb changes (Lee and Chan, 2023; Okahashi et al., 2014); for the low-difficulty ST, weak PFC activation in response to this cognitive load, coupled with low inter-individual variability, may mask the effects of the environmental intervention. The CNS assesses emotional and experiential bonds between humans and nature. Studies have demonstrated a positive correlation between CNS scores and mental health, and that greater connectedness to nature is associated with reduced fatigue and enhanced vitality (Capaldi et al., 2014). Our findings showing that CNS is negatively correlated with TSST (*r* = −0.435, *p* = 0.016) and MoCA with T-A (*r* = −0.435, *p* = 0.016) also corroborate this. While fNIRS data did not directly demonstrate enhanced cognitive ability in the forest group, a comprehensive analysis of their significantly higher CNS (*p* = 0.032) and MoCA (*p* < 0.001) scores compared with the urban group suggests that brief forest bathing can enhance natural connectedness and reduce cognitive fatigue.

**Figure 5:**
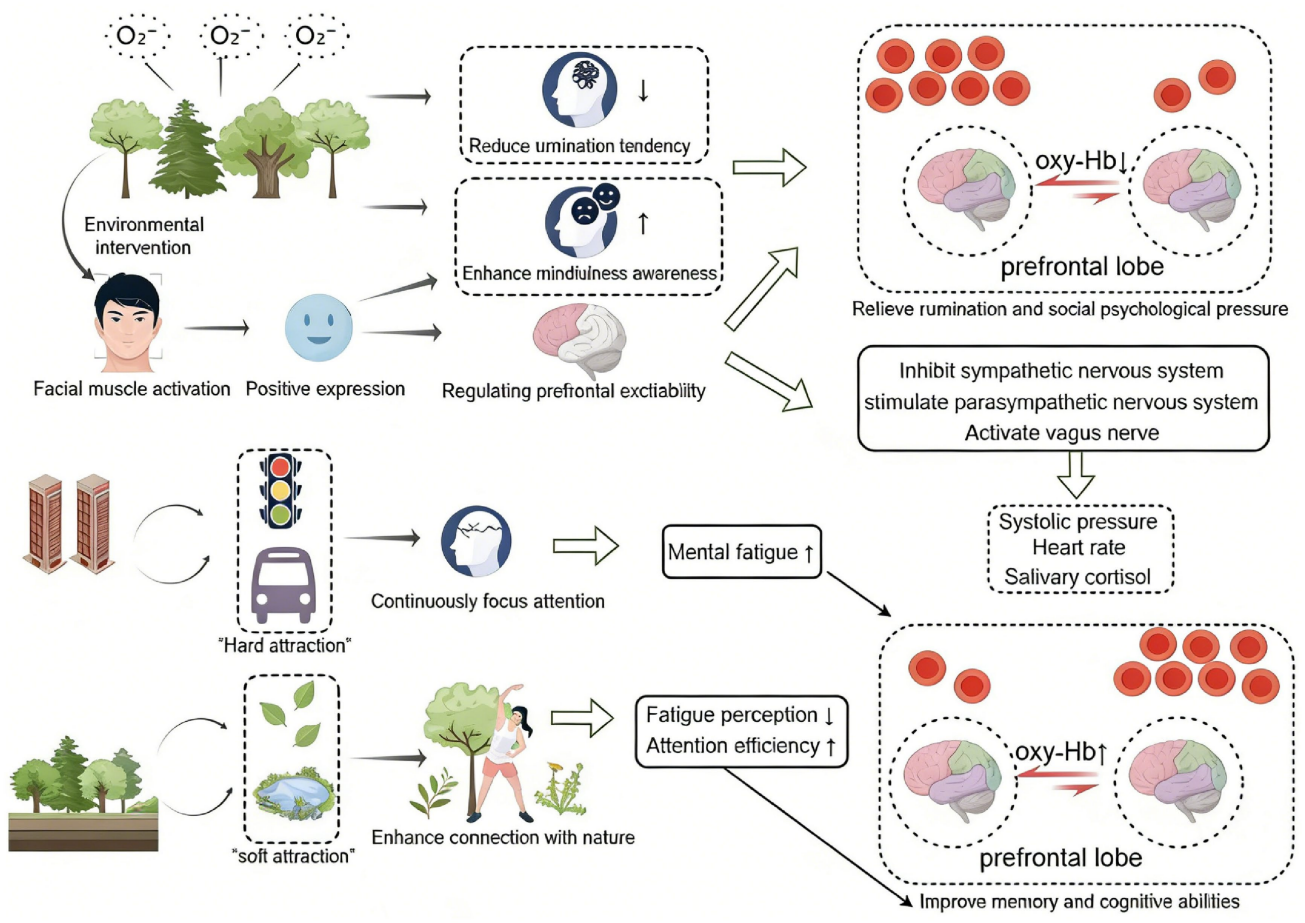
Mechanism underlying the effects of short-term forest bathing on undergraduates’ physical and mental health.

Blood pressure, heart rate, and salivary cortisol are key physiological markers for assessing an individual’s stress-related emotional state (Hakimi and Setarehdan, 2018). Green walking has been shown to lower blood pressure and heart rate (Ochiai et al., 2015; Wen et al., 2019). The current study found that participants in the forest environment had lower heart rate, systolic blood pressure, and salivary cortisol levels compared to those in the urban environment. This finding aligns with prior research on the cardiovascular benefits of natural environments (Ochiai et al., 2015; Takayama et al., 2022; Yau and Loke, 2020; Zeng et al., 2020). Moreover, research has linked cardiovascular recovery from stress to vagal activation (Mezzacappa et al., 2001). The present study hypothesizes that the effects of short-term forest bathing on blood pressure and heart rate may involve vagal excitation, alongside the parasympathetic stimulation and sympathetic inhibition mechanisms noted in earlier studies (Park et al., 2010; Queirolo et al., 2024; Wen et al., 2019). Forest environments outperform urban areas in sensory perception, atmosphere, and climate—factors that enhance the effectiveness of physiological interventions (Zeng et al., 2020). In this study, forests were found to be rich in NAI. Previous studies have reported that short-term exposure to forest NAI can stimulate the parasympathetic nervous system (Liu et al., 2022); specifically, NAI stimulates afferent nerves via nerve endings in the respiratory mucosa, triggering parasympathetic activation and acetylcholine release, which in turn induces cardiovascular relaxation effects (Jiang et al., 2018). These findings further support that brief forest bathing exerts positive effects on physical and mental health (see Figure 5).

However, no significant differences emerged in DBP changes. A substantial body of literature has documented DBP reductions after forest bathing (Mao et al., 2012; Ochiai et al., 2015; Park et al., 2010), but contradictory findings exist: some studies report no post-forest bathing DBP changes (Imamura et al., 2022; Lee et al., 2014), while systematic reviews/meta-analyses (macroscopic perspective) generally show declines in both SBP and DBP (Wen et al., 2019; Yau and Loke, 2020). This variability may stem from the multifaceted nature of DBP, which is influenced by numerous physiological and environmental factors. The current study’s reliance on self-reported data—particularly regarding gender—could introduce bias and limit generalizability (Kajantie and Phillips, 2006; Lee et al., 2014). Additionally, lifestyle factors such as physical activity and dietary habits may contribute to these variations (Seals et al., 2009). Specifically, oxygen saturation levels remained within the 97–98% range before and after walking in both groups, with no significant differences detected. While forest bathing has been shown to maintain high peripheral oxygen saturation (Lee et al., 2014), literature notes that among young individuals, changes in vascular oxygen saturation between 97 and 98% are negligible (Ribeiro et al., 2024). In this study, this pattern likely arises because participants already had high baseline oxygen saturation (98%) before walking, suggesting that short-term forest bathing may not have had a meaningful effect on oxygen saturation. Alternatively, this result could stem from natural variability or equipment error.

This study had several limitations: it was a single-center study with data derived exclusively from one institution, leading to limited generalizability; there was an imbalanced gender ratio (male:female = 7:53); indicators related to autonomic nervous system physiology were lacking; no follow-up component was implemented, precluding the evaluation of the durability of forest bathing effects; trait anxiety was not measured; PSS is primarily designed to assess overall stress levels over the preceding month, and thus may not be ideally suited for measuring short-term fluctuations within a single day; post-intervention, alternate versions were not used for psychological questionnaire measures, potentially leading to inflated test–retest effects that constitute a potential research bias; and cortisol may represent a potential confounding factor, as cortisol levels naturally decline throughout the day and are susceptible to the influence of metabolic status; references for the comfort index were from regionally indexed journals with limited international recognition.

## 5 Conclusion

Short-term forest bathing is an effective, low-cost stress reduction intervention for medical students, with easy implementation and no equipment needs. This study shows it reduces systolic blood pressure, heart rate and salivary cortisol; enhances nature connectedness; elevates positive emotions while diminishing negative ones; lowers cerebral blood flow to mitigate stress; and improves memory and reduce cognitive fatigue to a certain extent. In summary, this study preliminarily suggests that brief forest bathing exerts positive physiological and psychological effects on medical undergraduates. We recommend integrating it into mental health curricula with a theory-practice model to help medical students master sustainable stress management.

## Data Availability

The raw data supporting the conclusions of this article will be made available by the authors, without undue reservation.

## References

[ref1] Afrisham R. Sadegh-Nejadi S. SoliemaniFar O. Kooti W. Ashtary-Larky D. Alamiri F. . (2016). Salivary testosterone levels under psychological stress and its relationship with rumination and five personality traits in medical students. Psychiatry Investig. 13, 637–643. doi: 10.4306/pi.2016.13.6.637, 27909455 PMC5128352

[ref2] Aldao A. Nolen-Hoeksema S. Schweizer S. (2010). Emotion-regulation strategies across psychopathology: a meta-analytic review. Clin. Psychol. Rev. 30, 217–237. doi: 10.1016/j.cpr.2009.11.004, 20015584

[ref3] Antonelli M. Barbieri G. Donelli D. (2019). Effects of forest bathing (shinrin-yoku) on levels of cortisol as a stress biomarker: a systematic review and meta-analysis. Int. J. Biometeorol. 63, 1117–1134. doi: 10.1007/s00484-019-01717-x, 31001682

[ref4] Arnsten A. F. (2009). Stress signalling pathways that impair prefrontal cortex structure and function. Nat. Rev. Neurosci. 10, 410–422. doi: 10.1038/nrn2648, 19455173 PMC2907136

[ref5] Askim K. Knardahl S. (2021). The influence of affective state on subjective-report measurements: evidence from experimental manipulations of mood. Front. Psychol. 12:601083. doi: 10.3389/fpsyg.2021.601083, 33679520 PMC7930079

[ref6] Bang K. S. Lee I. Kim S. Lim C. S. Joh H. K. Park B. J. . (2017). The effects of a campus Forest-walking program on undergraduate and graduate students' physical and psychological health. Int. J. Environ. Res. Public Health 14:728. doi: 10.3390/ijerph14070728, 28678203 PMC5551166

[ref7] Bergmann C. Muth T. Loerbroks A. (2019). Medical students' perceptions of stress due to academic studies and its interrelationships with other domains of life: a qualitative study. Med. Educ. Online 24:1603526. doi: 10.1080/10872981.2019.1603526, 31007152 PMC6493308

[ref8] Boscolo A. Queirolo L. Navalesi P. (2025). The impact of psychophysiological well being on executive functions among anaesthesia residents. Eur. J. Anaesthesiol. 42, 366–368. doi: 10.1097/eja.0000000000002106, 39604253 PMC11872262

[ref9] Bratman G. N. Hamilton J. P. Hahn K. S. Daily G. C. Gross J. J. (2015). Nature experience reduces rumination and subgenual prefrontal cortex activation. Proc. Natl. Acad. Sci. USA 112, 8567–8572. doi: 10.1073/pnas.1510459112, 26124129 PMC4507237

[ref10] Bray I. Reece R. Sinnett D. Martin F. Hayward R. (2022). Exploring the role of exposure to green and blue spaces in preventing anxiety and depression among young people aged 14-24 years living in urban settings: a systematic review and conceptual framework. Environ. Res. 214:114081. doi: 10.1016/j.envres.2022.114081 35973463

[ref11] Bryan R. M. (1990). Cerebral blood flow and energy metabolism during stress. Am. J. Phys. 259, H269–H280. doi: 10.1152/ajpheart.1990.259.2.H269, 2167019

[ref12] Burrage E. Marshall K. L. Santanam N. Chantler P. D. (2018). Cerebrovascular dysfunction with stress and depression. Brain Circ 4, 43–53. doi: 10.4103/bc.bc_6_18, 30276336 PMC6126243

[ref13] Caetano I. Ferreira S. Coelho A. Amorim L. Castanho T. C. Portugal-Nunes C. . (2022). Perceived stress modulates the activity between the amygdala and the cortex. Mol. Psychiatry 27, 4939–4947. doi: 10.1038/s41380-022-01780-8, 36117211

[ref14] Capaldi C. A. Dopko R. L. Zelenski J. M. (2014). The relationship between nature connectedness and happiness: a meta-analysis. Front. Psychol. 5:976. doi: 10.3389/fpsyg.2014.00976, 25249992 PMC4157607

[ref15] Chen H. (2022) Characteristics of risk and moral decision – making in heroin addicts: a study combining functional near – infrared spectroscopy (fNIRS) [master's thesis]

[ref16] China Meteorological Administration (2017). Grade of air negative (oxygen) ion concentration. In (Vol. QX/T 380–2017). Beijing: China Meteorological Publishing House.

[ref17] de Faria D. D. Paulo A. J. M. Balardin J. Sato J. R. Junior E. A. Baltazar C. A. . (2020). Task-related brain activity and functional connectivity in upper limb dystonia: a functional magnetic resonance imaging (fMRI) and functional near-infrared spectroscopy (fNIRS) study. Neurophotonics 7:045004. doi: 10.1117/1.NPh.7.4.045004, 33094125 PMC7569470

[ref18] Fu G. Yu Y. Ye J. Zheng Y. Li W. Cui N. . (2023). A method for diagnosing depression: facial expression mimicry is evaluated by facial expression recognition. J. Affect. Disord. 323, 809–818. doi: 10.1016/j.jad.2022.12.029, 36535548

[ref19] General Administration of Quality Supervision, Inspection and Quarantine of the People’s Republic of China [AQSIQ] and Standardization Administration of the People’s Republic of China [SAC] (2008). Standard for environmental quality of sound (GB 3096–2008). In (Vol. GB 3096–2008). Beijing: China Standards Press.

[ref20] Hadanny A. Daniel-Kotovsky M. Suzin G. Boussi-Gross R. Catalogna M. Dagan K. . (2020). Cognitive enhancement of healthy older adults using hyperbaric oxygen: a randomized controlled trial. Aging 12, 13740–13761. doi: 10.18632/aging.103571, 32589613 PMC7377835

[ref21] Hakimi N. Setarehdan S. K. (2018). Stress assessment by means of heart rate derived from functional near-infrared spectroscopy. J. Biomed. Opt. 23, 1–12. doi: 10.1117/1.Jbo.23.11.115001, 30392197

[ref22] Hennenlotter A. Dresel C. Castrop F. Ceballos-Baumann A. O. Wohlschläger A. M. Haslinger B. (2009). The link between facial feedback and neural activity within central circuitries of emotion--new insights from botulinum toxin-induced denervation of frown muscles. Cereb. Cortex 19, 537–542. doi: 10.1093/cercor/bhn104, 18562330

[ref23] Herman J. P. (2012). Neural pathways of stress integration: relevance to alcohol abuse. Alcohol Res. 34, 441–447. doi: 10.35946/arcr.v34.4.08, 23584110 PMC3860392

[ref24] Horne M. J. Allbright M. Galbraith D. A. Patel A. (2024). Emotional intelligence in medicine: an investigation of the significance for physicians, residents, and medical students – a systematic review. J. Surg. Educ. 81:103307. doi: 10.1016/j.jsurg.2024.103307, 39471567

[ref25] Hussain A. Burdey D. M. B. (2023). Mediating role of emotional intelligence between the relationship of occupational stress and clinical performance among nurses. Bull. Bus. Econ. 12, 177–184. doi: 10.61506/01.00016

[ref26] Ikei H. Song C. Miyazaki Y. (2018). Physiological effects of touching the wood of Hinoki cypress (*Chamaecyparis obtusa*) with the soles of the feet. Int. J. Environ. Res. Public Health 15:2135. doi: 10.3390/ijerph15102135, 30274160 PMC6210085

[ref27] Imamura C. Sakakibara K. Arai K. Ohira H. Yamaguchi Y. Yamada H. (2022). Effect of indoor Forest bathing on reducing feelings of fatigue using cerebral activity as an Indicator. Int. J. Environ. Res. Public Health 19:6672. doi: 10.3390/ijerph19116672, 35682257 PMC9180409

[ref28] Int-Veen I. Fallgatter A. J. Ehlis A.-C. Rosenbaum D. (2023). Prefrontal hypoactivation induced via social stress is more strongly associated with state rumination than depressive symptomatology. Sci. Rep. 13:15147. doi: 10.1038/s41598-023-41403-y, 37704652 PMC10499935

[ref29] Jiang S. Y. Ma A. Ramachandran S. (2018). Negative air ions and their effects on human health and air quality improvement. Int. J. Mol. Sci. 19:2966. doi: 10.3390/ijms19102966, 30274196 PMC6213340

[ref30] Jimenez M. P. DeVille N. V. Elliott E. G. Schiff J. E. Wilt G. E. Hart J. E. . (2021). Associations between nature exposure and health: a review of the evidence. Int. J. Environ. Res. Public Health 18:4790. doi: 10.3390/ijerph18094790, 33946197 PMC8125471

[ref31] Jo H. Song C. Ikei H. Enomoto S. Kobayashi H. Miyazaki Y. (2019). Physiological and psychological effects of Forest and urban sounds using high-resolution sound sources. Int. J. Environ. Res. Public Health 16:2649. doi: 10.3390/ijerph16152649, 31344973 PMC6695879

[ref32] Kajantie E. Phillips D. I. (2006). The effects of sex and hormonal status on the physiological response to acute psychosocial stress. Psychoneuroendocrinology 31, 151–178. doi: 10.1016/j.psyneuen.2005.07.002, 16139959

[ref33] Kang B.-H. Shin W.-S. (2020). Forest therapy program reduces academic and job-seeking stress among college students. J. People Plants Environ. 23, 363–375. doi: 10.11628/ksppe.2020.23.3.363

[ref34] Kweon J. Kim Y. Choi H. Im W. Kim H. (2024). Enhancing sleep and reducing occupational stress through Forest therapy: a comparative study across job groups. Psychiatry Investig. 21, 1120–1128. doi: 10.30773/pi.2024.0118, 39465238 PMC11513866

[ref35] LeBlanc V. R. (2009). The effects of acute stress on performance: implications for health professions education. Acad. Med. 84, S25–S33. doi: 10.1097/ACM.0b013e3181b37b8f, 19907380

[ref36] Leblanc V. R. Regehr C. Tavares W. Scott A. K. Macdonald R. King K. (2012). The impact of stress on paramedic performance during simulated critical events. Prehosp. Disaster Med. 27, 369–374. doi: 10.1017/s1049023x12001021, 22831965

[ref37] Lee T. L. Chan A. S. (2023). Photobiomodulation may enhance cognitive efficiency in older adults: a functional near-infrared spectroscopy study. Front. Aging Neurosci. 15:1096361. doi: 10.3389/fnagi.2023.1096361, 37547747 PMC10397517

[ref38] Lee J. Tsunetsugu Y. Takayama N. Park B. J. Li Q. Song C. . (2014). Influence of forest therapy on cardiovascular relaxation in young adults. Evid. Based Complement. Alternat. Med. 2014:834360. doi: 10.1155/2014/834360, 24660018 PMC3934621

[ref39] Li Q. (2022). Effects of forest environment (Shinrin-yoku/Forest bathing) on health promotion and disease prevention -the establishment of “Forest medicine”. Environ. Health Prev. Med. 27:43. doi: 10.1265/ehpm.22-00160, 36328581 PMC9665958

[ref40] Li Q. Ochiai H. Ochiai T. Takayama N. Kumeda S. Miura T. . (2022). Effects of forest bathing (shinrin-yoku) on serotonin in serum, depressive symptoms and subjective sleep quality in middle-aged males. Environ. Health Prev. Med. 27:44. doi: 10.1265/ehpm.22-00136, 36328588 PMC9665960

[ref41] Liu S. Li C. Chu M. Zhang W. Wang W. Wang Y. . (2022). Associations of forest negative air ions exposure with cardiac autonomic nervous function and the related metabolic linkages: a repeated-measure panel study. Sci. Total Environ. 850:158019. doi: 10.1016/j.scitotenv.2022.158019, 35973547

[ref42] Lovati C. Manzi F. Di Dio C. Massaro D. Gilli G. Marchetti A. (2023). Feeling connected to nature: validation of the connectedness to nature scale in the Italian context. Front. Psychol. 14:1242699. doi: 10.3389/fpsyg.2023.1242699, 37901082 PMC10602663

[ref43] Lv J. Li X. Duan M. Meng X. Liu J. Zhao Y. . (2024). Analysis of microclimate and human comfort effect of riverside forest park in Shunyi District. Sci. Technol. Eng. 24, 7066–7072. doi: 10.12404/j.issn.1671-1815.2303641

[ref44] Macauley K. Plummer L. Bemis C. Brock G. Larson C. Spangler J. (2018). Prevalence and predictors of anxiety in healthcare professions students. Health Prof. Educ. 4, 176–185. doi: 10.1016/j.hpe.2018.01.001

[ref45] Macleod C. (1992). The Stroop task: the “gold standard” of attentional measures. J. Exp. Psychol. Gen. 121, 12–14. doi: 10.1037/0096-3445.121.1.12

[ref46] Mao G. X. Cao Y. B. Lan X. G. He Z. H. Chen Z. M. Wang Y. Z. . (2012). Therapeutic effect of forest bathing on human hypertension in the elderly. J. Cardiol. 60, 495–502. doi: 10.1016/j.jjcc.2012.08.003, 22948092

[ref47] Marselle M. R. Warber S. L. Irvine K. N. (2019). Growing resilience through interaction with nature: can group walks in nature buffer the effects of stressful life events on mental health? Int. J. Environ. Res. Public Health 16:986. doi: 10.3390/ijerph16060986, 30893850 PMC6466337

[ref48] Memon I. Omair A. Barradah O. M. Almegren N. M. Almuqbil M. M. Batarfi O. H. . (2023). Measurement of exam anxiety levels among medical students and their association with the influencing factors. Cureus 15:e41417. doi: 10.7759/cureus.41417, 37546066 PMC10403227

[ref49] Mezzacappa E. S. Kelsey R. M. Katkin E. S. Sloan R. P. (2001). Vagal rebound and recovery from psychological stress. Psychosom. Med. 63, 650–657. doi: 10.1097/00006842-200107000-00018, 11485119

[ref50] Morita E. Fukuda S. Nagano J. Hamajima N. Yamamoto H. Iwai Y. . (2007). Psychological effects of forest environments on healthy adults: shinrin-yoku (forest-air bathing, walking) as a possible method of stress reduction. Public Health 121, 54–63. doi: 10.1016/j.puhe.2006.05.024, 17055544

[ref51] Nasreddine Z. S. Phillips N. A. Bédirian V. Charbonneau S. Whitehead V. Collin I. . (2005). The Montreal cognitive assessment, MoCA: a brief screening tool for mild cognitive impairment. J. Am. Geriatr. Soc. 53, 695–699. doi: 10.1111/j.1532-5415.2005.53221.x 15817019

[ref52] Nejati V. Khorrami A. S. Fonoudi M. (2022). Neuromodulation of facial emotion recognition in health and disease: a systematic review. Neurophysiol. Clin. 52, 183–201. doi: 10.1016/j.neucli.2022.03.005, 35428551

[ref53] Ochiai H. Ikei H. Song C. Kobayashi M. Takamatsu A. Miura T. . (2015). Physiological and psychological effects of forest therapy on middle-aged males with high-normal blood pressure. Int. J. Environ. Res. Public Health 12, 2532–2542. doi: 10.3390/ijerph120302532, 25809507 PMC4377916

[ref54] Ohtani T. Matsuo K. Kasai K. Kato T. Kato N. (2009). Hemodynamic responses of eye movement desensitization and reprocessing in posttraumatic stress disorder. Neurosci. Res. 65, 375–383. doi: 10.1016/j.neures.2009.08.014 19729044

[ref55] Okahashi S. Mizumoto H. Komae A. Ueno K. Yokoyama M. Nagano A. . (2014). An fNIRS-based study on prefrontal cortex activity during a virtual shopping test with different task difficulties in brain-damaged patients. J. Behav. Brain Sci. 4, 247–255. doi: 10.4236/jbbs.2014.46026

[ref56] Ostrosky-Solís F. Lozano A. (2006). Digit span: effect of education and culture. Int. J. Psychol. 41, 333–341. doi: 10.1080/00207590500345724

[ref57] Park B. J. Tsunetsugu Y. Kasetani T. Kagawa T. Miyazaki Y. (2010). The physiological effects of Shinrin-yoku (taking in the forest atmosphere or forest bathing): evidence from field experiments in 24 forests across Japan. Environ. Health Prev. Med. 15, 18–26. doi: 10.1007/s12199-009-0086-9, 19568835 PMC2793346

[ref58] Pasanen T. Johnson K. Lee K. Korpela K. (2018). Can nature walks with psychological tasks improve mood, self-reported restoration, and sustained attention? Results from two experimental field studies. Front. Psychol. 9:2057. doi: 10.3389/fpsyg.2018.02057, 30425671 PMC6218585

[ref59] Pfeifer M. D. Scholkmann F. Labruyère R. (2018). Signal processing in functional near-infrared spectroscopy (fNIRS): methodological differences lead to different statistical results. Front. Hum. Neurosci. 11:641. doi: 10.3389/fnhum.2017.00641, 29358912 PMC5766679

[ref60] Pinti P. Tachtsidis I. Hamilton A. Hirsch J. Aichelburg C. Gilbert S. . (2020). The present and future use of functional near-infrared spectroscopy (fNIRS) for cognitive neuroscience. Ann. N. Y. Acad. Sci. 1464, 5–29. doi: 10.1111/nyas.13948, 30085354 PMC6367070

[ref61] Puxeddu M. G. Faskowitz J. Sporns O. Astolfi L. Betzel R. F. (2022). Multi-modal and multi-subject modular organization of human brain networks. NeuroImage 264:119673. doi: 10.1016/j.neuroimage.2022.119673 36257489

[ref62] Queirolo L. Fazia T. Roccon A. Pistollato E. Gatti L. Bernardinelli L. . (2024). Effects of forest bathing (Shinrin-yoku) in stressed people. Front. Psychol. 15:1458418. doi: 10.3389/fpsyg.2024.1458418, 39554703 PMC11565252

[ref63] Ramacciotti M. C. C. Soares Junior R. d. S. Sato J. R. Gualtieri M. (2024). Left OFC activation in functional near-infrared spectroscopy during an inhibitory control task in an early years sample: integrating stress responses with cognitive function and brain activation. Dev. Neurosci. 47, 81–97. doi: 10.1159/000539023 38663367 PMC11965844

[ref64] Ribeiro A. Soares R. Barbosa L. Silva A. Ferreira R. Terroso S. . (2024). Green environments and healthy aging: analyzing the role of green infrastructure in the functional well-being of seniors-a pilot study. Int. J. Environ. Res. Public Health 22:35. doi: 10.3390/ijerph22010035, 39857487 PMC11765493

[ref65] Rosenbaum D. Kroczek A. M. Hudak J. Rubel J. Maier M. J. Sorg T. . (2020). Neural correlates of mindful emotion regulation in high and low ruminators. Sci. Rep. 10:15617. doi: 10.1038/s41598-020-71952-5, 32973143 PMC7518445

[ref66] Seals D. R. Walker A. E. Pierce G. L. Lesniewski L. A. (2009). Habitual exercise and vascular ageing. J. Physiol. 587, 5541–5549. doi: 10.1113/jphysiol.2009.178822, 19723776 PMC2805366

[ref67] Söderkvist S. Ohlén K. Dimberg U. (2018). How the experience of emotion is modulated by facial feedback. J. Nonverbal Behav. 42, 129–151. doi: 10.1007/s10919-017-0264-1, 29497224 PMC5816132

[ref68] Song C. Ikei H. Kagawa T. Miyazaki Y. (2019). Effects of walking in a Forest on young women. Int. J. Environ. Res. Public Health 16:229. doi: 10.3390/ijerph16020229, 30650572 PMC6351942

[ref69] Song C. Ikei H. Miyazaki Y. (2014). Elucidation of the physiological adjustment effect of forest therapy. Nihon Eiseigaku Zasshi 69, 111–116. doi: 10.1265/jjh.69.111, 24858506

[ref70] Song C. Ikei H. Park B. J. Lee J. Kagawa T. Miyazaki Y. (2018). Psychological benefits of walking through forest areas. Int. J. Environ. Res. Public Health 15:2804. doi: 10.3390/ijerph15122804, 30544682 PMC6313311

[ref71] Strack F. Martin L. L. Stepper S. (1988). Inhibiting and facilitating conditions of the human smile: a nonobtrusive test of the facial feedback hypothesis. J. Pers. Soc. Psychol. 54, 768–777. doi: 10.1037//0022-3514.54.5.768 3379579

[ref72] Sudimac S. Sale V. Kühn S. (2022). How nature nurtures: amygdala activity decreases as the result of a one-hour walk in nature. Mol. Psychiatry 27, 4446–4452. doi: 10.1038/s41380-022-01720-6, 36059042 PMC9734043

[ref73] Takayama N. Morikawa T. Koga K. Miyazaki Y. Harada K. Fukumoto K. . (2022). Exploring the physiological and psychological effects of digital Shinrin-Yoku and its characteristics as a restorative environment. Int. J. Environ. Res. Public Health 19:1202. doi: 10.3390/ijerph19031202, 35162221 PMC8834905

[ref74] Tam A. Bateman S. Buckingham G. Wilson M. Melendez-Torres G. J. Vine S. . (2025). The effects of stress on surgical performance: a systematic review. Surg. Endosc. 39, 77–98. doi: 10.1007/s00464-024-11389-3, 39627555 PMC11666721

[ref75] Thomas C. L. Cassady J. C. (2021). Validation of the state version of the state-trait anxiety inventory in a university sample. SAGE Open 11, 1–10. doi: 10.1177/21582440211031900

[ref76] Trapnell P. D. Campbell J. D. (1999). Private self-consciousness and the five-factor model of personality: distinguishing rumination from reflection. J. Pers. Soc. Psychol. 76, 284–304. doi: 10.1037//0022-3514.76.2.284, 10074710

[ref77] Vermeesch A. L. Ellsworth-Kopkowski A. Prather J. G. Passel C. Rogers H. H. Hansen M. M. (2024). Shinrin-Yoku (Forest bathing): a scoping review of the global research on the effects of spending time in nature. Glob Adv Integr Med Health 13:27536130241231258. doi: 10.1177/27536130241231258, 38420597 PMC10901062

[ref78] Wang Y. J. Wang L. M. Ding Z. H. Qian C. Cao J. J. (2025). Effects of urban waterfront green space on summer microclimate. J. Nanjing For. Univ. 49, 233–241. doi: 10.12302/j.issn.1000-2006.202310003

[ref79] Wen Y. Yan Q. Pan Y. Gu X. Liu Y. (2019). Medical empirical research on forest bathing (Shinrin-yoku): a systematic review. Environ. Health Prev. Med. 24:70. doi: 10.1186/s12199-019-0822-8, 31787069 PMC6886167

[ref80] Westlund Schreiner M. Roberts H. Dillahunt A. K. Farstead B. Feldman D. Thomas L. . (2023). Negative association between non-suicidal self-injury in adolescents and default mode network activation during the distraction blocks of a rumination task. Suicide Life Threat. Behav. 53, 510–521. doi: 10.1111/sltb.12960, 36942887 PMC10441767

[ref81] Wilcox T. Biondi M. (2015). fNIRS in the developmental sciences. Wiley Interdiscip. Rev. Cogn. Sci. 6, 263–283. doi: 10.1002/wcs.1343, 26263229 PMC4979552

[ref82] Woo E. Sansing L. H. Arnsten A. F. T. Datta D. (2021). Chronic stress weakens connectivity in the prefrontal cortex: architectural and molecular changes. Chron Stress 5:24705470211029254. doi: 10.1177/24705470211029254, 34485797 PMC8408896

[ref83] Xi C. E. Boet S. Assi A. Sikora L. McConnell M. M. (2025). Influence of emotions on clinical performance in acute care: a scoping review. PLoS One 20:e0329445. doi: 10.1371/journal.pone.0329445, 40758697 PMC12321073

[ref84] Xu Y. G. H. Barbour R. (2016). “nirsLAB user manual” in Neuroimaging informatics tools and resources clearinghouse (NITRC). Bethesda, MD, USA. Available online at: https://www.nitrc.org/projects/fnirs_downstate

[ref85] Yamaoka K. Uotsu N. Hoshino E. (2022). Relationship between psychosocial stress-induced prefrontal cortex activity and gut microbiota in healthy participants-a functional near-infrared spectroscopy study. Neurobiol. Stress 20:100479. doi: 10.1016/j.ynstr.2022.100479, 36039149 PMC9418982

[ref86] Yamashita R. Chen C. Matsubara T. Hagiwara K. Inamura M. Aga K. . (2021). The mood-improving effect of viewing images of nature and its neural substrate. Int. J. Environ. Res. Public Health 18:5500. doi: 10.3390/ijerph18105500, 34065588 PMC8161053

[ref87] Yau K. K. Loke A. Y. (2020). Effects of forest bathing on pre-hypertensive and hypertensive adults: a review of the literature. Environ. Health Prev. Med. 25:23. doi: 10.1186/s12199-020-00856-7, 32571202 PMC7310560

[ref88] Yılmaz Koğar E. Koğar H. (2024). A systematic review and meta-analytic confirmatory factor analysis of the perceived stress scale (PSS-10 and PSS-14). Stress. Health 40:e3285. doi: 10.1002/smi.3285, 37341705

[ref89] Zeng C. Lyu B. Deng S. Yu Y. Li N. Lin W. . (2020). Benefits of a three-day bamboo forest therapy session on the physiological responses of university students. Int. J. Environ. Res. Public Health 17:3238. doi: 10.3390/ijerph17093238, 32384727 PMC7246605

[ref90] Zhang M. Wu J. Yang Y. Song J. Shen Q. (2025). Mediating role of emotional intelligence in the relationship between dual work stress and reflective ability among junior nurses. BMC Nurs. 24:547. doi: 10.1186/s12912-025-03178-7, 40380165 PMC12082875

[ref91] Zhu B. (1995). Brief introduction to the POMS scale and its short-form Chinese norm. J. Tianjin Inst. Phys. Educ. 10, 36–37. doi: 10.13297/j.cnki.issn1005-0000.1995.01.007

