# Supplementary material for: Exploring the effects of short-term forest bathing on anxious medical undergraduates’ stressful emotions using near-infrared functional brain imaging and facial expression technology
Source: Front Psychol. 2026 Mar 11;17:1734650. doi: 10.3389/fpsyg.2026.1734650 (PMC13013367; doi:10.3389/fpsyg.2026.1734650)
Supplement: Supplementary file 1 [file Supplementary_file_1.docx]

Supplementary Material 1: Brain regions corresponding to each channel of fNIRS

Supplementary Table S1 Location of fNIRS channels corresponding to prefrontal brain areas

| Prefrontal Brain Region | Channel |
| --- | --- |
| Dorsolateral Prefrontal Cortex  Frontopolar Area | CH2、CH5、CH8、CH9、CH10、CH15、CH17  CH6、CH7、CH12、CH14、CH16 |
| Ventrolateral Prefrontal Cortex | CH1、CH3、CH18、CH20 |
| Orbitofrontal Cortex | CH4、CH11、CH13、CH19 |

Supplementary Material 2: fNIRS Task-Specific Protocols

①RT: The task displays six thought-provoking questions, each shown for five seconds, prompting participants to reflect on the respective question.

②MT: Five sets of letters are presented in this task, each appearing for 5 seconds with a sequential increase in letter count. Participants are directed to read each set of letters aloud in order as they appear on the screen.

③ST: This task presents 12 colored words; participants are asked to quickly identify the color of each word.

④TSST: A speech topic is displayed on the screen for this task. Participants receive 20 seconds to prepare, followed by a 1-minute speech delivery.

**Data Recording Duration and Temporal Window Definitions**

Two fNIRS recording sessions (pre- and post-intervention) were conducted with identical protocols, each lasting 19 min 29 s (1169 s): signal stabilization-Empty—run to ensure fNIRS signal stability before task initiation (1 min), RT task (40 s), eye-closed relaxation (30 s), TSST task (80 s: 20-s preparation + 1-min speech), eye-closed relaxation (30 s), MT task (35 s), eye-closed relaxation (30 s), and ST task (84 s), with a 2-s red "+" cue inserted between trials of all tasks to indicate upcoming presentations. The interval between the two sessions was a 2-hour intervention.

**Baseline window:** Defined as the 30-s eye-closed relaxation period immediately preceding each task. This period was selected for its low cognitive load (no task engagement) and minimal interference (eye-closed to reduce visual activation, relaxation to minimize motion artifacts).

**Post-intervention window:** Corresponding functional segments of each task in the post-intervention session, ensuring direct comparability with pre-intervention data.

HbO concentration averages were computed via fixed task blocks averaging: For the RT task (6×5-s thinking segments), MT task (5×5-s letter reading segments), TSST task (1-min speech segment), and ST task (12×5-s color identification segment), HbO values of task-specific functional periods were extracted and averaged to reflect task-related physiological responses.

Supplementary Material 3: Lu Dinghuang Composite Comfort Index

The formula is: S=0.6(|T-24|) + 0.07(|RH-70|) + 0.5(|V-2|) (1)

In formula (1), S represents the composite comfort index; T denotes temperature (℃); RH stands for relative humidity (%); and V indicates average wind speed (m/s).

Supplementary Table S2 Comfort Index Evaluation Criteria

| Level | Range | Comfort Level |
| --- | --- | --- |
| 1 | S≤4.55 | Comfortable |
| 2 | 4.55≤S≤6.95 | Relatively Comfortable |
| 3 | 6.95≤S≤9.00 | Uncomfortable |
| 4 | S＞9.00 | Extremely Uncomfortable |

Supplementary Material 4: Air negative (oxygen) ion concentration grades defined in the *Grade of Air Negative (Oxygen) Ion Concentration* (QX/T 380-2017) and Daytime ambient noise limits specified in the *Environmental Quality Standard for Noise* (GB 3096-2008).

Supplementary Table S3 Concentration Levels of Negative Oxygen Ions in Air

| Level | air negative ion concentration (N) | explanation |
| --- | --- | --- |
| I | N≥1200 | High concentration, fresh air |
| II | 500≤N<1200 | Relatively high concentration, relatively fresh air |
| III | 100≤N<500 | Medium concentration, moderate air quality |
| IV | 0≤N<100 | Low concentration, insufficiently fresh air |

Supplementary Table S4 Ambient Noise Limits

| Functional area category | Daytime (dB(A)) |
| --- | --- |
| Category 0 (e.g., rehabilitation and convalescent areas) | 50 |
| Category 1 (e.g. residential dwellings) | 55 |
| Category 2 (e.g., market trade areas) | 60 |
| Category 3 (e.g., industrial generation zones) | 65 |
| Category 4a (e.g. highways) | 70 |
| Category 4b (e.g., areas on both sides of a main railroad line) | 70 |

Supplementary Material 5: Demographic Characteristics of Participants

| Supplementary Table S5 Demographic Characteristics of Participants | | | | | | |
| --- | --- | --- | --- | --- | --- | --- |
| Index | Forest Group^①^(n=30) | | | City Group^①^(n=30) | *U*/*t*/*χ^2^* | *P* |
| Age | 19.800±0.997 | 19.467±0.937 | | | 374.000^②^ | 0.238 |
| Sex |  |  | | | 0.162^④^ | 0.688 |
| Male | 4(57.1%) | 3(42.9%) | | |  |  |
| Female | 26(49.1%) | 27(50.9%) | | |  |  |
| Height | 158.150±6.751 | | 160.000±5.852 | | -0.766^③^ | 0.447 |
| Weight | 53.133±7.615 | | 51.833±6.406 | | 0.716^③^ | 0.477 |
| Spirometry | 2352.933±610.870 | | 2317.800±591.363 | | 0.226^③^ | 0.822 |
| Smoking status |  | |  | | 0.268^④^ | 0.605 |
| Positive | 1(25.0%) | | 3(75.0%) | |  |  |
| Negative | 29(51.8%) | | 27(48.2%) | |  |  |
| Drinking status |  | |  | | 1.364^④^ | 0.243 |
| Positive | 10(62.5%) | | 6(37.5%) | |  |  |
| Negative | 20(45.5%) | | 24(54.5%) | |  |  |
| Campaign situation |  | |  | | 5.815^④^ | 0.121 |
| 0 times | 5(83.3%) | | 1(16.7%) | |  |  |
| 1~2 times | 20(54.1%) | | 17(45.9%) | |  |  |
| 3~4 times | 4(28.6%) | | 10(71.4%) | |  |  |
| more than 5 times | 1(33.3%) | | 2(66.7%) | |  |  |
| STAI-S | 41.100±8.510 | | 40.033±8.927 | | 0.429^③^ | 0.669 |

#①Data is expressed as mean±standard deviation or number of people (%); ②means *U*-value；③means *t*-value；④means *χ^2^*-value.

Supplementary Material 6: Effects of Short-Term Forest Bathing on Specific Cerebral Channels Across Tasks

Notable variations in cerebral oxy-Hb concentrations were observed across brain channels during task performance.

**TSST:** Pre- to post-forest bathing, the forest group showed significant oxy-Hb fluctuations across 8 channels (CH2, CH3, CH4, CH6, CH7, CH11, CH13, CH16)—spanning the DLPFC, FPC, OFC, and VLPFC—whereas the urban group had no significant oxy-Hb changes in these channels (see Table S6).

**MT:** No significant oxy-Hb differences were found between the forest group and adjacent channels; however, the urban group exhibited significant oxy-Hb increases in channels linked to the DLPFC, FPA, and OFC (CH14, CH15, CH17, CH19).

Supplementary Table S6 Specific Changes in Oxyhemoglobin Concentration in Effective Channels under Different Tasks

| Task | Channel | Before(μM) | After(μM) | *t* | *P*(FDR adjusted) |
| --- | --- | --- | --- | --- | --- |
| TSST | 2^①^ | 0.126±0.206 | 0.019±0.150 | -2.490 | 0.047* |
|  | 3^①^ | -0.064±0.306 | -0.221±0.252 | -2.611 | 0.041* |
|  | 4^①^ | 0.031±0.313 | -0.133±0.275 | -2.638 | 0.041* |
|  | 6^①^ | -0.154±0.244 | -0.327±0.269 | -2.892 | 0.036* |
|  | 7^①^ | 0.037±0.280 | -0.149±0.277 | -2.987 | 0.036* |
|  | 11^①^ | 0.011±0.183 | -0.114±0.193 | -2.603 | 0.041* |
|  | 13^①^ | 0.071±0.174 | -0.097±0.182 | -3.271 | 0.036* |
|  | 16^①^ | -0.024±0.201 | -0.192±0.184 | -3.114 | 0.036* |
| MT | 14^②^ | 0.009±0.106 | 0.080±0.101 | 3.267 | 0.019* |
|  | 15^②^ | 0.016±0.081 | 0.076±0.079 | 3.876 | 0.006** |
|  | 17^②^ | 0.016±0.082 | 0.062±0.076 | 3.887 | 0.006** |
|  | 19^②^ | -0.018±0.130 | 0.061±0.116 | 2.968 | 0.030* |

#①means forest group ②means city group **P*<0.05, **0.001 ≤ *P*<0.01

Supplementary Material 7: Further correlation analysis was performed among the forest group for pre-to-post changes in aggregated subject-level oxy-Hb (fNIRS tasks), physiological indicators, and scale scores, as well as post-intervention facial expression data (See Table S7).

Supplementary Table S7: Results of Hybrid Correlation Analyses for Experimental Indices in the Forest Group

| **Variable1** | **Variable2** | **Method** | ***r*-effect** | ***P*** | **Significance** |
| --- | --- | --- | --- | --- | --- |
| Fe | PSS | spearman | -0.797 | <0.001 | *** |
| TDM | F-I | pearson | 0.684 | <0.001 | *** |
| TDM | D-D | pearson | 0.643 | <0.001 | *** |
| DSB | C-B | pearson | -0.539 | 0.002 | ** |
| TDM | T-A | pearson | 0.492 | 0.006 | ** |
| F-I | D-D | pearson | 0.485 | 0.007 | ** |
| MT | S-E | spearman | 0.48 | 0.007 | ** |
| TDM | C-B | pearson | 0.475 | 0.008 | ** |
| SC | RRQ-I | pearson | 0.468 | 0.009 | ** |
| TDM | V-A | pearson | -0.462 | 0.010 | * |
| TDM | A-H | pearson | 0.457 | 0.011 | * |
| TSST | CNS | pearson | -0.435 | 0.016 | * |
| MoCA | T-A | pearson | -0.435 | 0.016 | * |
| SPO2 | RRQ-I | pearson | -0.424 | 0.020 | * |
| DBP | HR | pearson | 0.416 | 0.022 | * |
| DSB | MoCA | pearson | 0.41 | 0.025 | * |
| TDM | S-E | pearson | -0.408 | 0.025 | * |
| DSB | T-A | pearson | -0.399 | 0.029 | * |
| MT | HR | spearman | -0.393 | 0.032 | * |
| MT | ST | spearman | 0.39 | 0.033 | * |
| Fe | V-A | spearman | 0.388 | 0.034 | * |
| TSST | DSB | pearson | 0.386 | 0.035 | * |
| MT | DBP | spearman | -0.382 | 0.037 | * |
| RRQ-R | TDM | pearson | -0.381 | 0.038 | * |
| SC | SPO2 | pearson | -0.37 | 0.044 | * |
| Fe | A-H | spearman | -0.369 | 0.045 | * |
| T-A | D-D | pearson | 0.368 | 0.045 | * |
| SBP | T-A | pearson | 0.366 | 0.047 | * |
| DSB | TDM | pearson | -0.363 | 0.048 | * |
| RRQ-I | S-E | pearson | -0.36 | 0.051 | ns |
| DBP | C-B | pearson | 0.354 | 0.055 | ns |
| A-H | D-D | pearson | 0.351 | 0.057 | ns |
| SBP | DBP | pearson | 0.351 | 0.057 | ns |
| SPO2 | F-I | pearson | -0.348 | 0.060 | ns |
| PSS | A-H | pearson | 0.341 | 0.065 | ns |
| CNS | DSB | pearson | -0.338 | 0.068 | ns |
| ST | HR | spearman | -0.335 | 0.070 | ns |
| HR | SC | pearson | 0.331 | 0.074 | ns |
| Fe | TDM | spearman | -0.322 | 0.083 | ns |
| RT | V-A | pearson | -0.319 | 0.086 | ns |
| SBP | CNS | pearson | -0.317 | 0.087 | ns |
| SC | S-E | pearson | 0.315 | 0.090 | ns |
| SBP | MoCA | pearson | -0.306 | 0.100 | ns |
| SPO2 | D-D | pearson | -0.305 | 0.101 | ns |
| ST | RRQ-I | spearman | -0.289 | 0.122 | ns |
| CNS | V-A | pearson | -0.286 | 0.126 | ns |
| A-H | F-I | pearson | 0.285 | 0.128 | ns |
| RT | D-D | pearson | -0.281 | 0.132 | ns |
| ST | F-I | spearman | -0.28 | 0.133 | ns |
| PSS | TDM | pearson | 0.279 | 0.136 | ns |
| CNS | A-H | pearson | -0.275 | 0.142 | ns |
| PSS | V-A | pearson | -0.274 | 0.143 | ns |
| RRQ-R | S-E | pearson | 0.273 | 0.145 | ns |
| RRQ-I | RRQ-R | pearson | -0.272 | 0.146 | ns |
| F-I | S-E | pearson | -0.27 | 0.149 | ns |
| ST | DBP | spearman | -0.269 | 0.151 | ns |
| CNS | MoCA | pearson | -0.259 | 0.167 | ns |
| RRQ-R | A-H | pearson | -0.259 | 0.168 | ns |
| ST | RT | spearman | 0.258 | 0.168 | ns |
| MT | TDM | spearman | -0.25 | 0.184 | ns |
| TSST | HR | pearson | 0.249 | 0.184 | ns |
| CNS | C-B | pearson | 0.248 | 0.186 | ns |
| TSST | C-B | pearson | -0.246 | 0.190 | ns |
| SBP | A-H | pearson | 0.244 | 0.195 | ns |
| Fe | F-I | spearman | -0.243 | 0.196 | ns |
| RRQ-R | D-D | pearson | -0.243 | 0.197 | ns |
| TSST | SBP | pearson | 0.241 | 0.199 | ns |
| HR | F-I | pearson | 0.238 | 0.206 | ns |
| RRQ-I | TDM | pearson | 0.236 | 0.210 | ns |
| ST | A-H | spearman | 0.234 | 0.214 | ns |
| SC | V-A | pearson | -0.233 | 0.215 | ns |
| ST | SBP | spearman | 0.229 | 0.224 | ns |
| RRQ-I | D-D | pearson | 0.229 | 0.224 | ns |
| SPO2 | CNS | pearson | -0.22 | 0.243 | ns |
| RRQ-R | V-A | pearson | 0.219 | 0.244 | ns |
| MoCA | TDM | pearson | -0.219 | 0.246 | ns |
| D-D | C-B | pearson | 0.218 | 0.247 | ns |
| RT | F-I | pearson | -0.218 | 0.248 | ns |
| RRQ-R | MoCA | pearson | 0.214 | 0.256 | ns |
| MT | RRQ-I | spearman | -0.212 | 0.260 | ns |
| SPO2 | TDM | pearson | -0.21 | 0.265 | ns |
| DBP | TDM | pearson | 0.209 | 0.267 | ns |
| DBP | CNS | pearson | 0.208 | 0.270 | ns |
| RT | C-B | pearson | 0.207 | 0.273 | ns |
| HR | V-A | pearson | -0.203 | 0.283 | ns |
| DBP | RRQ-R | pearson | 0.202 | 0.284 | ns |
| SBP | HR | pearson | 0.202 | 0.285 | ns |
| MT | SBP | spearman | -0.199 | 0.291 | ns |
| DSB | D-D | pearson | -0.199 | 0.292 | ns |
| SPO2 | RRQ-R | pearson | 0.198 | 0.294 | ns |
| SC | D-D | pearson | 0.198 | 0.294 | ns |
| PSS | D-D | pearson | 0.197 | 0.296 | ns |
| HR | RRQ-R | pearson | -0.195 | 0.301 | ns |
| TSST | F-I | pearson | -0.195 | 0.301 | ns |
| HR | TDM | pearson | 0.195 | 0.301 | ns |
| RT | DSB | pearson | -0.192 | 0.309 | ns |
| TSST | D-D | pearson | -0.19 | 0.314 | ns |
| SBP | TDM | pearson | 0.189 | 0.317 | ns |
| V-A | C-B | pearson | -0.188 | 0.319 | ns |
| ST | V-A | spearman | -0.187 | 0.324 | ns |
| RT | MoCA | pearson | 0.182 | 0.335 | ns |
| TSST | TDM | pearson | -0.18 | 0.342 | ns |
| ST | RRQ-R | spearman | -0.18 | 0.342 | ns |
| RT | T-A | pearson | -0.179 | 0.344 | ns |
| MoCA | V-A | pearson | 0.178 | 0.347 | ns |
| F-I | V-A | pearson | -0.178 | 0.347 | ns |
| RRQ-I | T-A | pearson | 0.176 | 0.353 | ns |
| ST | MoCA | spearman | -0.175 | 0.354 | ns |
| PSS | T-A | pearson | 0.175 | 0.356 | ns |
| MT | RT | spearman | -0.172 | 0.362 | ns |
| TSST | A-H | pearson | 0.172 | 0.363 | ns |
| MT | T-A | spearman | -0.171 | 0.367 | ns |
| T-A | F-I | pearson | 0.171 | 0.367 | ns |
| DSB | V-A | pearson | 0.17 | 0.370 | ns |
| T-A | S-E | pearson | -0.169 | 0.371 | ns |
| ST | T-A | spearman | 0.167 | 0.378 | ns |
| RRQ-R | F-I | pearson | -0.166 | 0.380 | ns |
| MoCA | D-D | pearson | -0.164 | 0.387 | ns |
| ST | S-E | spearman | 0.163 | 0.388 | ns |
| PSS | C-B | pearson | -0.163 | 0.390 | ns |
| RT | A-H | pearson | 0.162 | 0.394 | ns |
| Fe | T-A | spearman | -0.158 | 0.404 | ns |
| Fe | SBP | spearman | -0.157 | 0.409 | ns |
| Fe | DSB | spearman | -0.152 | 0.422 | ns |
| TSST | DBP | pearson | 0.152 | 0.424 | ns |
| RT | SBP | pearson | 0.149 | 0.433 | ns |
| TSST | MoCA | pearson | 0.146 | 0.441 | ns |
| ST | SC | spearman | -0.146 | 0.441 | ns |
| ST | Fe | spearman | -0.144 | 0.448 | ns |
| T-A | C-B | pearson | 0.143 | 0.452 | ns |
| RRQ-I | V-A | pearson | -0.141 | 0.459 | ns |
| SPO2 | V-A | pearson | 0.14 | 0.460 | ns |
| RRQ-I | MoCA | pearson | -0.138 | 0.468 | ns |
| ST | D-D | spearman | -0.136 | 0.472 | ns |
| DBP | S-E | pearson | -0.136 | 0.473 | ns |
| TSST | RRQ-I | pearson | 0.135 | 0.476 | ns |
| RRQ-R | DSB | pearson | 0.132 | 0.485 | ns |
| MoCA | A-H | pearson | 0.13 | 0.492 | ns |
| PSS | RRQ-R | pearson | -0.13 | 0.493 | ns |
| ST | PSS | spearman | 0.129 | 0.497 | ns |
| TSST | PSS | pearson | -0.128 | 0.500 | ns |
| RT | RRQ-I | pearson | 0.128 | 0.501 | ns |
| TSST | SC | pearson | 0.125 | 0.509 | ns |
| Fe | D-D | spearman | -0.125 | 0.510 | ns |
| DSB | F-I | pearson | -0.125 | 0.510 | ns |
| HR | SPO2 | pearson | -0.125 | 0.510 | ns |
| MT | A-H | spearman | -0.125 | 0.512 | ns |
| SC | MoCA | pearson | 0.123 | 0.517 | ns |
| F-I | C-B | pearson | 0.123 | 0.519 | ns |
| HR | RRQ-I | pearson | 0.121 | 0.524 | ns |
| RT | HR | pearson | -0.118 | 0.533 | ns |
| MT | F-I | spearman | -0.117 | 0.537 | ns |
| MT | PSS | spearman | -0.116 | 0.542 | ns |
| CNS | PSS | pearson | 0.115 | 0.546 | ns |
| ST | CNS | spearman | -0.115 | 0.546 | ns |
| PSS | F-I | pearson | 0.114 | 0.548 | ns |
| ST | TSST | spearman | 0.114 | 0.548 | ns |
| MoCA | S-E | pearson | 0.113 | 0.552 | ns |
| RRQ-I | F-I | pearson | 0.112 | 0.557 | ns |
| CNS | RRQ-R | pearson | -0.108 | 0.570 | ns |
| PSS | MoCA | pearson | -0.107 | 0.573 | ns |
| T-A | A-H | pearson | 0.107 | 0.574 | ns |
| SPO2 | DSB | pearson | -0.106 | 0.578 | ns |
| RRQ-I | A-H | pearson | -0.106 | 0.578 | ns |
| RRQ-R | C-B | pearson | -0.106 | 0.578 | ns |
| MT | SPO2 | spearman | -0.105 | 0.581 | ns |
| SBP | C-B | pearson | -0.102 | 0.592 | ns |
| MT | RRQ-R | spearman | 0.099 | 0.601 | ns |
| CNS | F-I | pearson | -0.098 | 0.605 | ns |
| DSB | A-H | pearson | 0.097 | 0.609 | ns |
| C-B | S-E | pearson | -0.096 | 0.612 | ns |
| SBP | S-E | pearson | -0.096 | 0.614 | ns |
| SBP | PSS | pearson | 0.095 | 0.617 | ns |
| MoCA | F-I | pearson | -0.095 | 0.617 | ns |
| TSST | V-A | pearson | 0.095 | 0.617 | ns |
| SBP | F-I | pearson | 0.095 | 0.617 | ns |
| RT | DBP | pearson | 0.094 | 0.621 | ns |
| RRQ-R | T-A | pearson | -0.092 | 0.629 | ns |
| DBP | SC | pearson | -0.091 | 0.632 | ns |
| SC | A-H | pearson | -0.089 | 0.639 | ns |
| TSST | T-A | pearson | -0.089 | 0.640 | ns |
| A-H | S-E | pearson | 0.089 | 0.641 | ns |
| SBP | RRQ-R | pearson | 0.089 | 0.641 | ns |
| MT | C-B | spearman | -0.088 | 0.644 | ns |
| SC | CNS | pearson | -0.088 | 0.645 | ns |
| DBP | V-A | pearson | -0.087 | 0.649 | ns |
| HR | S-E | pearson | -0.086 | 0.651 | ns |
| HR | C-B | pearson | 0.086 | 0.652 | ns |
| RT | TSST | pearson | -0.085 | 0.657 | ns |
| CNS | D-D | pearson | 0.084 | 0.660 | ns |
| CNS | S-E | pearson | 0.083 | 0.663 | ns |
| SPO2 | S-E | pearson | -0.082 | 0.665 | ns |
| SPO2 | MoCA | pearson | -0.08 | 0.673 | ns |
| SBP | RRQ-I | pearson | 0.079 | 0.680 | ns |
| SPO2 | C-B | pearson | 0.078 | 0.680 | ns |
| HR | D-D | pearson | 0.078 | 0.683 | ns |
| SC | PSS | pearson | -0.077 | 0.687 | ns |
| MoCA | C-B | pearson | 0.075 | 0.692 | ns |
| SC | T-A | pearson | 0.073 | 0.700 | ns |
| ST | DSB | spearman | -0.073 | 0.702 | ns |
| DBP | T-A | pearson | 0.073 | 0.703 | ns |
| SBP | DSB | pearson | -0.07 | 0.713 | ns |
| MT | MoCA | spearman | -0.07 | 0.713 | ns |
| ST | SPO2 | spearman | -0.068 | 0.721 | ns |
| Fe | HR | spearman | 0.068 | 0.721 | ns |
| RRQ-I | C-B | pearson | -0.068 | 0.723 | ns |
| CNS | TDM | pearson | 0.067 | 0.724 | ns |
| HR | MoCA | pearson | 0.067 | 0.725 | ns |
| SPO2 | T-A | pearson | -0.066 | 0.729 | ns |
| TSST | RRQ-R | pearson | -0.065 | 0.733 | ns |
| HR | DSB | pearson | -0.063 | 0.739 | ns |
| T-A | V-A | pearson | 0.063 | 0.741 | ns |
| DBP | D-D | pearson | 0.061 | 0.751 | ns |
| Fe | DBP | spearman | 0.057 | 0.764 | ns |
| SPO2 | A-H | pearson | -0.055 | 0.775 | ns |
| MT | Fe | spearman | 0.054 | 0.776 | ns |
| V-A | S-E | pearson | 0.054 | 0.778 | ns |
| Fe | C-B | spearman | 0.053 | 0.779 | ns |
| SC | F-I | pearson | -0.053 | 0.781 | ns |
| SC | DSB | pearson | -0.052 | 0.783 | ns |
| RT | CNS | pearson | 0.051 | 0.789 | ns |
| SC | RRQ-R | pearson | 0.051 | 0.791 | ns |
| Fe | CNS | spearman | 0.05 | 0.792 | ns |
| DBP | F-I | pearson | 0.048 | 0.801 | ns |
| DBP | SPO2 | pearson | 0.047 | 0.805 | ns |
| A-H | V-A | pearson | -0.046 | 0.809 | ns |
| Fe | TSST | spearman | 0.045 | 0.813 | ns |
| DBP | MoCA | pearson | -0.045 | 0.814 | ns |
| Fe | SPO2 | spearman | -0.045 | 0.815 | ns |
| ST | TDM | spearman | 0.043 | 0.821 | ns |
| PSS | RRQ-I | pearson | 0.041 | 0.829 | ns |
| Fe | RRQ-I | spearman | -0.039 | 0.837 | ns |
| CNS | RRQ-I | pearson | 0.039 | 0.840 | ns |
| SPO2 | PSS | pearson | 0.037 | 0.848 | ns |
| PSS | DSB | pearson | 0.036 | 0.849 | ns |
| SBP | SC | pearson | 0.036 | 0.852 | ns |
| RT | SC | pearson | 0.035 | 0.853 | ns |
| DBP | DSB | pearson | -0.035 | 0.855 | ns |
| ST | C-B | spearman | -0.03 | 0.876 | ns |
| RT | TDM | pearson | 0.029 | 0.878 | ns |
| DBP | PSS | pearson | -0.029 | 0.880 | ns |
| HR | CNS | pearson | 0.029 | 0.881 | ns |
| Fe | S-E | spearman | 0.028 | 0.883 | ns |
| MT | DSB | spearman | -0.027 | 0.888 | ns |
| RT | SPO2 | pearson | -0.027 | 0.889 | ns |
| DBP | RRQ-I | pearson | 0.026 | 0.893 | ns |
| RT | PSS | pearson | -0.025 | 0.896 | ns |
| MT | D-D | spearman | 0.025 | 0.897 | ns |
| Fe | MoCA | spearman | -0.023 | 0.902 | ns |
| SC | TDM | pearson | 0.022 | 0.907 | ns |
| SC | C-B | pearson | -0.021 | 0.914 | ns |
| A-H | C-B | pearson | -0.019 | 0.921 | ns |
| CNS | T-A | pearson | 0.019 | 0.921 | ns |
| HR | A-H | pearson | -0.018 | 0.925 | ns |
| TSST | S-E | pearson | 0.017 | 0.927 | ns |
| PSS | S-E | pearson | -0.016 | 0.935 | ns |
| D-D | V-A | pearson | -0.015 | 0.936 | ns |
| MT | TSST | spearman | 0.015 | 0.936 | ns |
| MT | CNS | spearman | 0.015 | 0.936 | ns |
| HR | PSS | pearson | -0.013 | 0.944 | ns |
| SBP | SPO2 | pearson | 0.013 | 0.945 | ns |
| RT | S-E | pearson | 0.012 | 0.948 | ns |
| MT | SC | spearman | 0.011 | 0.953 | ns |
| Fe | RT | spearman | 0.01 | 0.956 | ns |
| SBP | V-A | pearson | -0.008 | 0.966 | ns |
| Fe | RRQ-R | spearman | 0.008 | 0.967 | ns |
| DBP | A-H | pearson | 0.007 | 0.971 | ns |
| RRQ-I | DSB | pearson | -0.007 | 0.971 | ns |
| DSB | S-E | pearson | 0.005 | 0.980 | ns |
| TSST | SPO2 | pearson | -0.004 | 0.982 | ns |
| D-D | S-E | pearson | 0.004 | 0.985 | ns |
| RT | RRQ-R | pearson | 0.003 | 0.986 | ns |
| SBP | D-D | pearson | -0.003 | 0.989 | ns |
| HR | T-A | pearson | -0.002 | 0.991 | ns |
| Fe | SC | spearman | 0.001 | 0.995 | ns |
| MT | V-A | spearman | <0.001 | 0.998 | ns |

* The sample size was 30 for both groups; Fe means Facial expression; **P*<0.05, **0.001≤*P*<0.01 ****P*<0.001


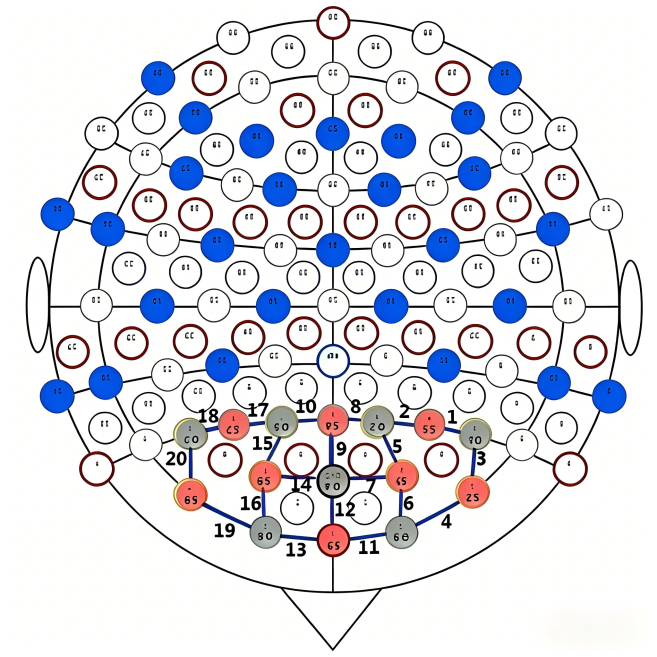


Supplementary Fig S1 fNIRS Light Source, Detector, and Channel Distribution


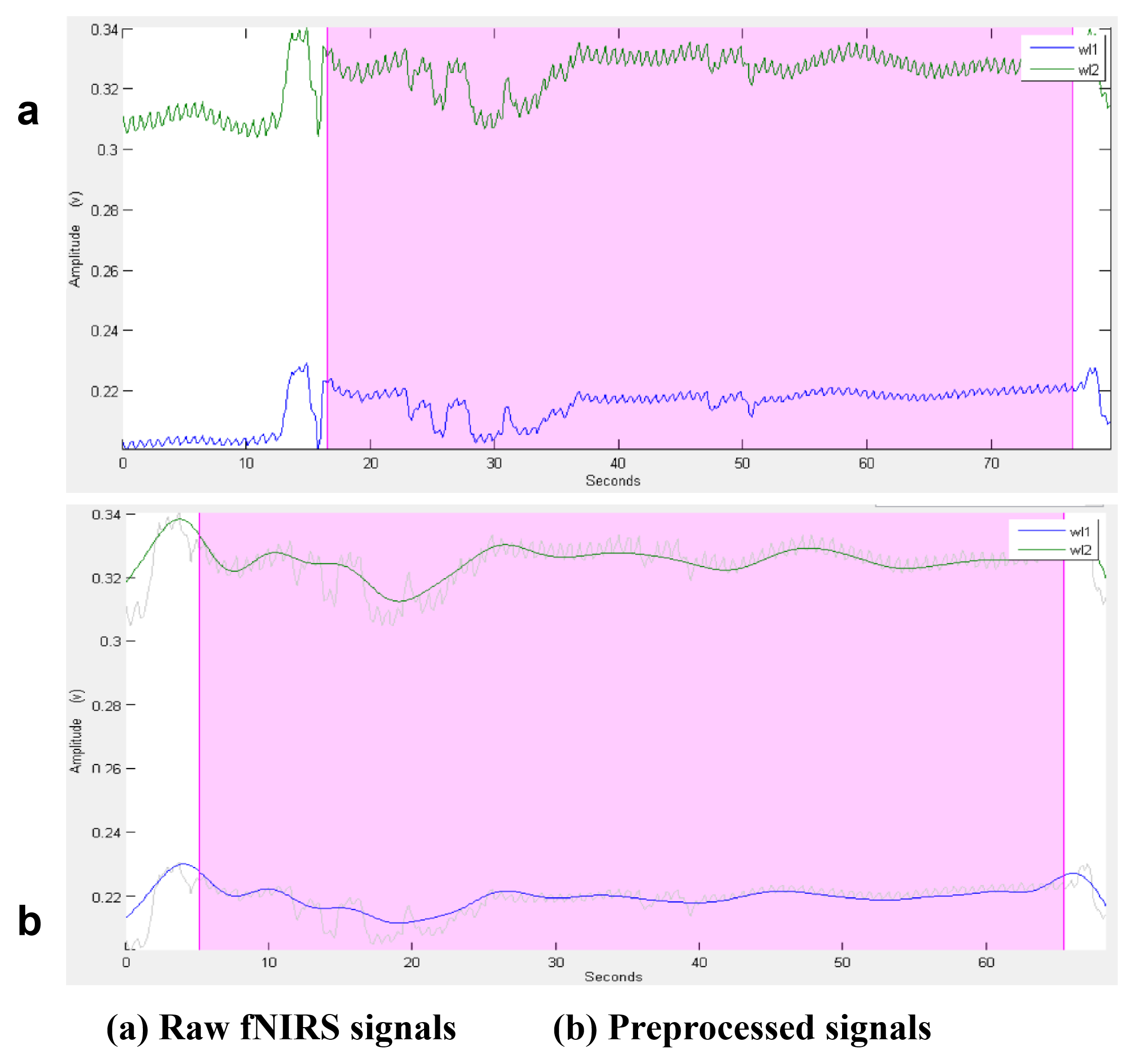


Supplementary Fig S2 Comparison of raw and preprocessed signals (a) Raw fNIRS signals; (b) Preprocessed signals.


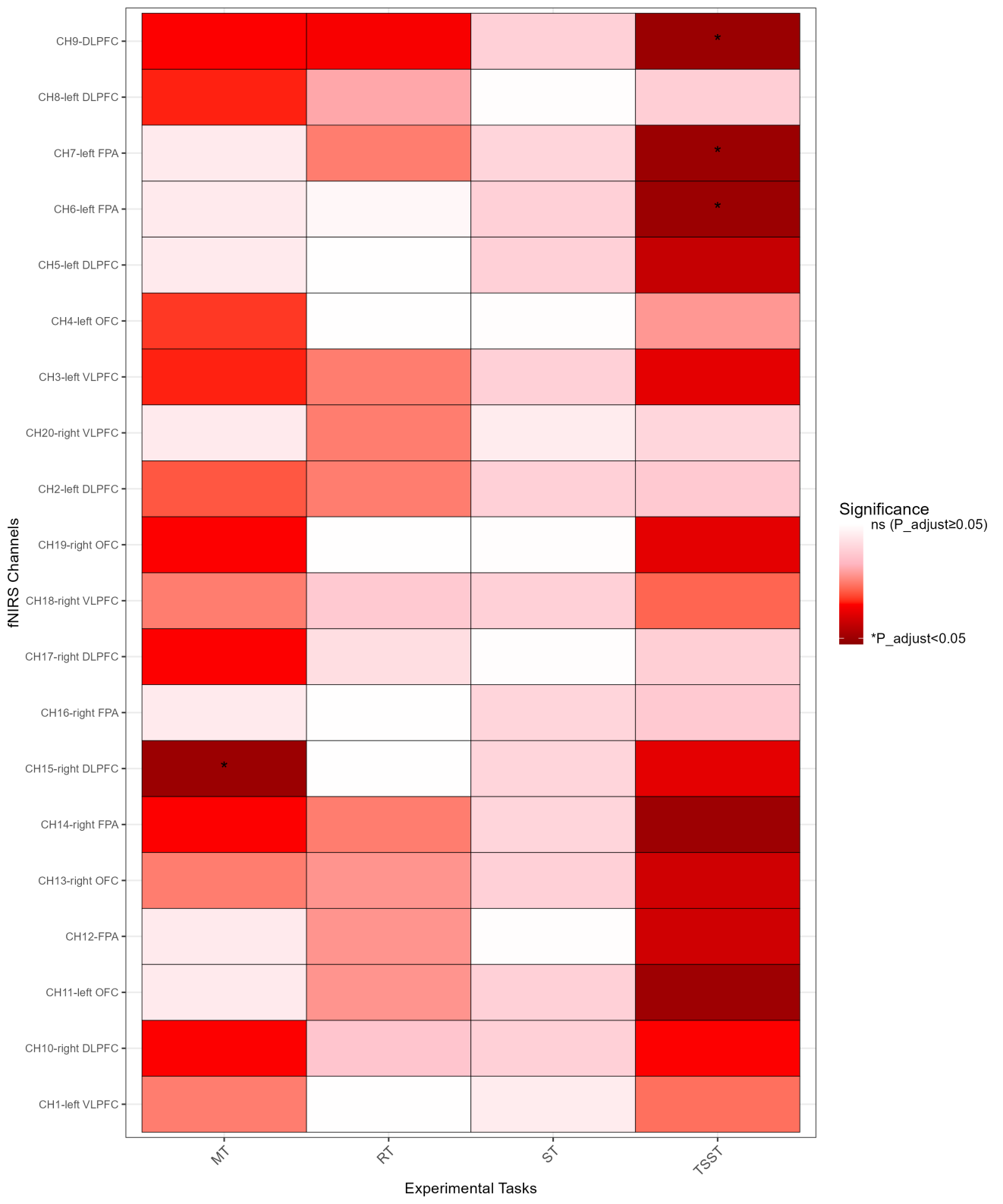


Supplementary Fig S3 Heatmap of Difference Significance in Pre-Post Oxy-Hb Delta Values for Specific fNIRS Channels Between Two Groups Across Four Tasks *P_*adjust means P_adjust denotes the P-value corrected via the false discovery rate (FDR). * indicates a significant difference in pre-post oxy-Hb delta values between the two groups (*P_*adjust < 0.05).
